# The Expression and Prognostic Value of FGF2, FGFR3, and FGFBP1 in Esophageal Squamous Cell Carcinoma

**DOI:** 10.1155/2020/2872479

**Published:** 2020-12-11

**Authors:** Wenjing Zhang, Yaxing Zhou, Chao Li, Shanshan Xu, Mengyan Li, Wenying Liu, Yuqing Ma, Hui Wang

**Affiliations:** ^1^Department of Pathology, First Affiliated Hospital, Xinjiang Medical University, Urumqi, Xinjiang, China; ^2^Department of RICU, First Affiliated Hospital, Xinjiang Medical University, Urumqi, Xinjiang, China; ^3^Department of Oncology, First Affiliated Hospital, Xinjiang Medical University, Urumqi, Xinjiang, China

## Abstract

**Background:**

Esophageal squamous cell carcinoma was treated by operation and chemoradiotherapy. However, the prognosis of most patients is poor after treatment, and most studies have shown that FGF2 and its receptor (FGFR) are involved in the development of various malignant tumors. FGF2 plays an important role in tumor progression and malignancy. In this study, the immunohistochemistry of FGF2, FGFR3, and FGFBP1 was used to further verify the expression of the three proteins in 172 patients with esophageal squamous cell carcinoma (ESCC) who had not received preoperative chemoradiotherapy and its effect on the prognosis of ESCC.

**Methods:**

(1) *χ*^2^ test was used to analyze the relationship between proteins and clinicopathological parameters. Survival analysis was used to investigate the effect of three proteins on prognosis. (2) Paired sample *t*-test was used to analyze the mRNA expression of the three proteins in fresh ESCC tissues and adjacent normal tissues.

**Results:**

FGF2 was correlated with tumor size (*p* = 0.026), gender (*p* = 0.047), and lymph metastasis (*p* = 0.007) in ESCC tissues. The high expression of FGFR3 was associated with tumor differentiation (*p* = 0.043 and *p* < 0.05), lymph node metastasis (*p* = 0.078 and *p* < 0.1), and race (*p* = 0.033 and *p* < 0.05). The high expression of FGFBP1 was significantly associated with the degree of tumor differentiation (*p* = 0.012), age (*p* = 0.045), and lymph node metastasis (*p* = 0.032) of ESCC patients. The expression of FGF2, FGFR3, and FGFBP1-mRNA in ESCC tissues was significantly higher than that in adjacent tissues (*p* < 0.001, *p* < 0.001, and *p* = 0.001). Patients with high expression of FGF2, FGFBP1, and FGFR3 had poor prognosis. There was a weak positive correlation between FGF2 and FGFBP1, as well as FGFR.

**Conclusion:**

The FGF2-FGFR3 axis may promote the progression of esophageal squamous cell carcinoma. The FGF2-FGFR3 axis may be a new direction of targeted therapy for esophageal squamous cell carcinoma. FGF2 and FGFR3 may be used as prognostic markers of esophageal squamous cell carcinoma.

## 1. Introduction

Esophageal cancer is a common digestive tract malignant tumor, which in sorted by histological type includes esophageal squamous cell carcinoma and esophageal adenocarcinoma. In European and American countries, the pattern is approximately 70% Barrett esophageal adenocarcinomas; while in China, the pattern is 95% esophageal squamous cell carcinoma [1], of which the 5-year survival rate is only 5%–13% [2]. There are obvious regional and national differences in the distribution of esophageal cancer in China. The high-incidence area of esophageal cancer is mainly distributed in the north China area, Dabie Mountain area, and between Fujian and Guangdong coastal area, and Xinjiang is also one of the high-risk areas [3]. The mechanism that occurs in the development of esophageal cancer is a complex combination with interactions at multistage, of multiple factors, and between multiple genes [4, 5]. Treatment of esophageal cancer should be the use of surgery and chemoradiotherapy; however, due to the late detection of most patients, the prognosis is poor after treatment.

Growth factor fibroblast growth factor-2 (FGF2), also known as basic FGF [6], has been shown to exist in low molecular and high molecular weight isomers that are translated by a single common mRNA through another translation initiation codon [7]. Low molecular weight FGF2 is an 18 kDa protein translated from the traditional AUG initiation codon. LMW FGF2, which exists in cytoplasm and nucleus, can also be secreted by the target cells. In order to start signal, the compound of LMW, FGF2, cell surface heparin sulfate proteoglycans (HSPGs), and fibroblast growth factor receptor (FGFR) activates downstream signaling pathways, including Ras, Raf, MAPK, and ERK [8]. The high molecular weight (HMW) FGF2 subtype is initiated by the translation of the upstream CUG locus and AUG codon frame. HMW FGF2 was located in the nucleus, and the signal was independent of FGFR [9]. The downstream signaling pathways are mediated by Ras/ERK and phosphoribosyl kinase (PI3K)/AKT signaling pathways in order to promote cell mitosis and regulate cell proliferation, differentiation, and migration [10]. FGF2 also plays an important role in tumor progression and malignancy, such as breast cancer and oral squamous cell carcinoma. FGF2, regulating CSCs through Mek/Erk signaling, is an important factor in esophageal squamous cell carcinoma [11].

FGFR, FGF2 receptors, is made of three Ig-like domains in the extracellular region, a single spanning transmembrane domain, and a split tyrosine kinase domain in the cytoplasmic region [12]. As FGF binds to FGFRs, the tyrosine kinase domain in the cytoplasmic region of the receptors is activated and generates signal paths, such as the Ras-MAPK, PI3K-AKT, and PLC-*γ*-PKC pathways to induce cell proliferation, differentiation, migration, and tumor formation [13]. FGFR3 has carcinogenic activity in several cancers. The increased or mutated expression in FGFR3 leads to malignant progression in bladder cancer, colon cancer, and multiple myeloma [14–17].

FGFBP1 can bind to fibroblast growth factors such as FGF2, protect FGF2 from degradation, and present it to its high-affinity cell surface receptor, thus promoting the biological function of FGFs. FGFBP1 was reversibly combined with the acidic and basic fibroblast growth factor. FGF2 can closely bind HSPG in ECM and is only released through the action of FGFBP1 [18]. FGF binding protein is the key to FGF bioavailability regulatory factors [19]. Increasingly study has shown that FGFBP1 is highly expressed in skin cancer [20, 21], but not clear in the mechanism of esophageal cancer.

The FGF/FGFR tyrosine kinase signal pathway regulates multiple biological events during embryogenesis and functions in the maintenance and repair of adult tissues [22]. This pathway is also implicated in both tumorigenesis and the development of chemoresistance in various types of cancers [23].

This study is designed to preliminarily analyze the expression of FGF2, FGFR3, FGFBP1, and their relationships with clinicopathological parameters in ESCC. The correlation among these proteins was analyzed through Spearman correlation analysis. Furthermore, we investigated to evaluate the effect of FGF2, FGFR3, and FGFBP1 on the prognosis of ESCC through Kaplan–Meier analysis. Our findings suggested that the FGF2-FGFR3 axis may be a new direction of targeted therapy for ESCC.

## 2. Materials and Methods

From January 2014 to June 2018, 172 cases of esophageal squamous cell carcinoma (including 94 cases of Han nationality and 78 cases of Hazak nationality) were collected from the First Affiliated Hospital of Xinjiang Medical University, including their clinicopathological data: age (<65 and ≥65), gender (male and female), nationality, location (upper, middle, and lower), tumor size (<3 cm and ≥3 cm), differentiation degree (high differentiation, middle differentiation, and low differentiation), lymph node metastasis (yes and no), vascular infiltration (yes and no), nerve infiltration (yes and no); distant metastasis (yes and no), pTNM stage (the eighth edition) (IB, IIA+B, and IIIA+B+C), and treatment (surgery and postoperative chemoradiotherapy) (Table 1). None of the selected patients completed neoadjuvant therapy preoperatively. All the selected patients were patients with advanced esophageal squamous cell carcinoma without early cancer (T1N0M0). All the selected patients underwent radical resection of esophageal carcinoma and lymph node dissection. According to 2020 CSCO esophagus cancer diagnosis and treatment guidelines, the patients of cT1b cT2 N+ or cT3-cT4a, any N needs to be radical surgery and at the same time chemoradiotherapy. All the selected patients in this study required postoperative chemoradiotherapy. And the study was approved by Ethical Committee of the First Affiliated Hospital of Xinjiang Medical University. Our follow-up time ended in July 2019 through inquiring the medical records and telephone calls.

### 2.1. Immunohistochemistry

(1) Anti-FGFR3 (product no. BM5016), anti-FGF2 antibody (EP1735) (ab92337), and anti-FGFBP1 antibody (ab238155) were used as the reagents

(2) *Methods*: with SP method, 172 cases of embedded esophageal cancer, paraffin tissue, and normal mucosal tissue were made into tissue chips, which were made into 4 mic continuous sections. The tissues were surgical samples. After dewaxing and dehydration, the tissue chips were put into a boiling repair solution, citric acid (PH6.0), heated to 95°C, and kept them in the acid for 20 minutes. After 30-minute cooling at room temperature, these chips were added into endogenous peroxidase and incubated for 20 minutes in the room temperature. The tissue chips were washed in phosphate-buffered saline for three-times (3 min/time). After that, anti-FGFR3 (BM5016) (1 : 50, 4°C overnight), anti-FGFBP1 antibody (ab238155) (1 : 800, 4°C overnight), and anti-FGF2 antibody (EP1735) (ab92337) (1 : 800, 4°C overnight) were dipped onto these chips separately. When the time is up, the chips were washed in phosphate buffer saline (PBS) for three times, were dropped with goat anti-mouse secondary antibody (PV-6002, Zsbio, Beijing, China), and were placed in an oven at 37°C for 40 minutes. Finally, the slides were dyed in prepared DAB solution, redyed with hematoxylin, dehydrated with graded alcohol, covered with slide, and installed for review.

(3) The staining intensity score of FGFBP1 was 0 (negative), 1 (weak), and 2 (strong). The dyeing range is based on the percentage of positive tumor cell score of 0 (negative), 1 (1% and 25%), 2 (26% and 50%), 3 (51% and 75%), or 4 (76% and 100%). The final score is the product of the staining intensity score and the staining range score. If the final score is 0 to 4(±), the case is ultimately considered negative. If the final score is 5(+) to 8(+++), the final score is positive. Expression levels of FGF2 and FGFR3 were assessed by semiquantitative scoring, including percentage of total lesion area (0-100%) and staining intensity (0-3). The expression of epithelium, endothelial cells, and stroma was analyzed in all cases. The classification of area positive rate is as follows: <10% = 0, 10‐25% = 1, 25‐50% = 2, 50‐75% = 3, and >75% = 4. To assess intensity, the grades were as follows: 0: none; 1: mild; 2: moderate; and 3: strong staining. The percentage score (0–4) was multiplied by the intensity score (0–3), and the final score was assigned with 0–4 for negative staining and 5–12 for positive staining [24]

### 2.2. qRT-PCR

(1) *Extraction of total RNA*: firstly, 29 cases of esophageal cancer and their paired adjacent normal tissues were taken out from the refrigerator at -80°C. Secondly, the liquid nitrogen was added to them for milling, and Trizol reagent was also added to extract the total RNA in the tissues after grinding according to the instructions. Thirdly, the concentration of total RNA in the extracted tissues was measured by NanoDrop 2000c uv spectrophotometer. After the electrophoresis test, cDNA was transcribed. According to the instructions of the reverse transcription kit, 2 ng taken from the total RNA extracted was added into the reverse transcription reaction system under the following conditions: 25°C 5 min, 42°C 60 min, 70°C 5 min, and 4°C forever. The synthesized cDNA was stored in a refrigerator at -80°C for later use. The primer sequences are listed in Table 2.

(2) qRT-PCR use a two-step method with SYBR Green (Applied Biosystems 7500, Thermo Fisher Scientific)

(3) The above reaction elements were added into the reaction system, and the reaction conditions were as follows: predenaturation at 95°C for 2 min, denaturation at 95°C for 10 s, annealing at 60°C for 10 s, extension at 72°C for 5 min, and 40 cycles in total. Each sample was repeated at least 3 times, with 3 multiple holes set for each time (Table 2)

### 2.3. Statistical Analysis

SPSS 25.0 statistical software was used. *χ*^2^ test and Fisher exact test were used to analyze the relationship between clinicopathological characteristics and the expression of FGF2, FGFR3, and FGFBP1 in esophageal squamous cell carcinoma. The correlation between FGF2, FGFR3, and FGFBP1 was also analyzed by Spearman grade correlation. Use overall survival and progression-free survival to estimate survival time. Progression-free survival is defined as the diagnosis of esophageal squamous cell carcinoma of the time to tumor progression or death. Overall survival is defined as the diagnosis of esophageal cancer patients with time to death or final follow-up time (2019-07-01). The effects of FGF2, FGFR3, FGFBP1 protein, and clinicopathological parameters on the prognosis of ESCC were analyzed by Kaplan–Meier method. Based on Kaplan–Meier analysis results, independent factors related to the prognosis of esophageal squamous cell carcinoma were further analyzed by Cox proportional risk model. The method was forward LR, and *p* < 0.05 was considered significant. For qRT-PCR results, the ct value of the cancerous tissue and the ct value of the adjacent tissues were used to calculate the 2-*ΔΔ*ct value. If the two groups of values accord with normal distribution and homogeneity of variance, the paired sample *t*-test is adopted. If the measured data are nonnormal and homogeneity of variance, the nonparametric Wilcoxon rank sum test is adopted.

## 3. Results and Analysis

### 3.1. The Expression of FGF2, FGFR3, and FGFBP1 in ESCC and Their Relationship with Clinicopathological Parameters

**Figure 1:**
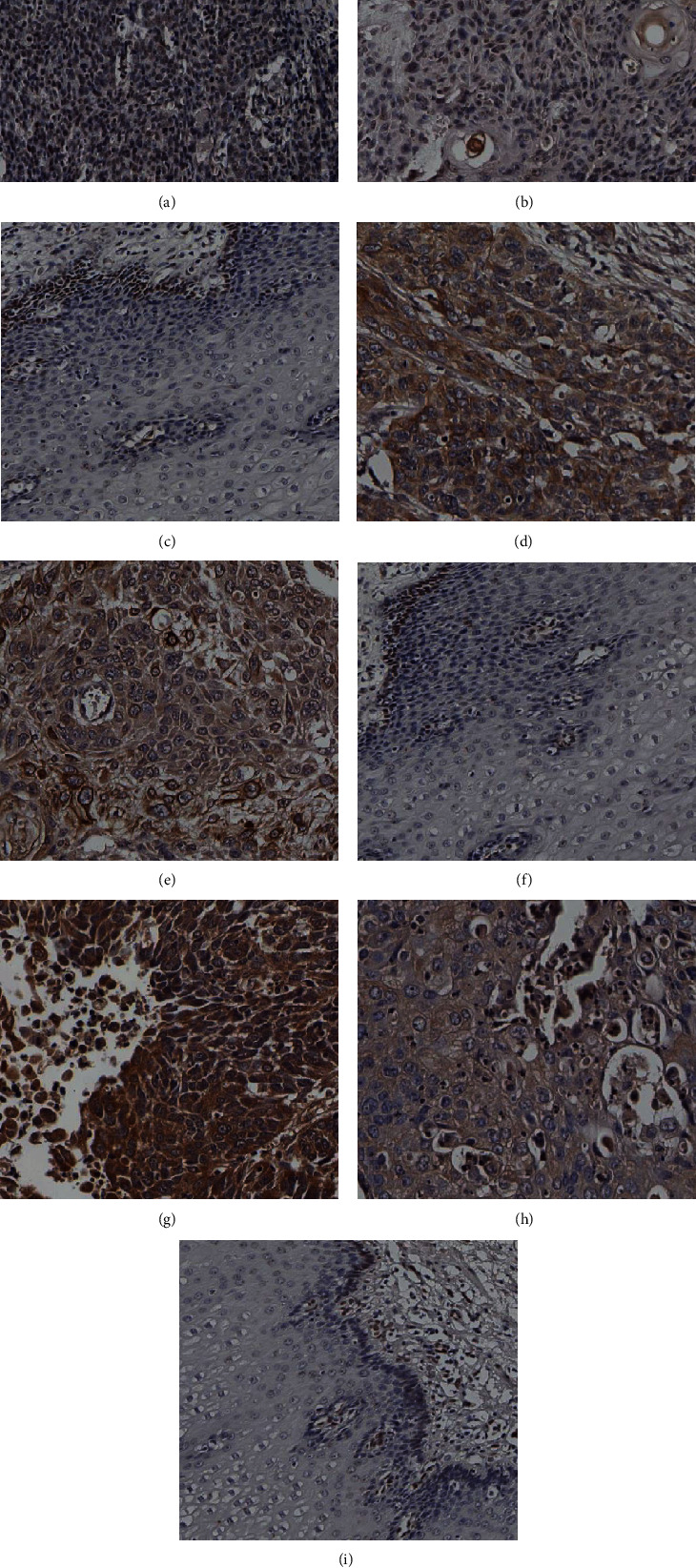
Immunohistochemical staining of FGF2, FGFR3, FGFBP1 expression in ESCC and normal esophagus mucosa. (a, b) Positive expression of FGF2 in ESCC tumor tissue; (c) positive expression of FGF2 in the basal layer of normal esophageal mucosa; (d, e) positive expression of FGFR3 in ESCC tumor tissue; (f) positive expression of FGFR3 in the basal layer of normal esophageal mucosa; (g, h) positive expression of FGFBP1 in ESCC tumor tissue; (i) negative expression of FGFBP1 in normal esophageal mucosa.

The expression of FGF2 in ESCC is shown in the figure (Figures 1(a)–1(c)). FGF2 was positive in the nucleus and cytoplasm of ESCC, negative in normal esophageal mucosa, or positive only in basal cells. In this study, there were 172 patients with esophageal squamous cell carcinoma, of which 55 (32%) were FGF2 negative and 117 (68%) were FGF2 positive. Statistical analysis showed that high expression of FGF2 was correlated with tumor size (*p* = 0.026), gender (*p* = 0.047), and lymph metastasis (*p* = 0.007). The expression of FGF2 was not correlated with race (*p* = 0.794), age (*p* = 0.053), tumor site (*p* = 0.902), differentiation (*p* = 0.231), and pathological stage of ESCC cases (*p* = 0.325) (*p* > 0.05) and so on (Table 3).

The expression of FGFR3 in ESCC and its relationship with clinicopathological parameters was investigated. As is shown in Figures 1(d)–1(f), positive FGFR3 staining signals were brown and yellow, located in the cytoplasm and membrane of the esophageal cancer cells. In normal tissues adjacent to cancer, FGFR3 positive signals were found in the basal layer of esophageal mucosa. All tumor specimens were divided into the FGFR3-low expression group (61 cases, 35.5%) and the FGFR3-high expression group (111 cases, 64.5%). As is shown in Table 3 summary, the correlation is between FGFR3 expression and clinicopathological features. Statistical analysis showed that overexpression of FGFR3 expression was correlated with tumor differentiation (*p* = 0.043 and *p* < 0.05), lymph node metastasis (*p* = 0.078 and *p* < 0.1), and race (*p* = 0.033 and *p* < 0.05).

The expression of FGFBP1 in ESCC is shown in Figures 1(g)–1(i). The positive signal of FGFBP1 is mainly located in the cytoplasm and membrane of the esophageal cancer cells and is positively expressed in the normal esophageal mucosal epithelium. Statistical analysis showed that the high expression of FGFBP1 was significantly correlated with the degree of tumor differentiation (*p* = 0.012), age (*p* = 0.045), and lymph node metastasis (*p* = 0.032) of ESCC patients, while no association was significantly correlated for high expression of FGFBP1 with gender (*p* = 0.559), race (*p* = 0.302), tumor size (*p* = 0.267), tumor site (*p* = 0.457), pathological stage (*p* = 0.320), vascular invasion (*p* = 0.735), and so on (*p* > 0.05) (Table 3).

### 3.2. Correlation of FGF2, FGFR3, and FGFBP1 Protein Expression

Spearman level correlation analysis was performed in 172 cases of esophageal squamous cell carcinoma, and the protein expression of FGF2 was significantly correlated with FGFR3 and FGFBP1 (*p* < 0.001, rs = 0.612; *p* < 0.001, rs = 0.649). FGFR3 and FGFBP1 were further analyzed and found that there is a positive correlation (*p* < 0.001, rs = 0.656), as is shown in Tables 4 and 5. These three proteins are highly expressed in esophageal squamous cell carcinoma. According to their correlation, it was speculated that the FGF2-FGFR3 axis formed by FGF2, FGFR3, and FGFBP1 may promote ESCC progression.

### 3.3. qRT-PCR

**Figure 2:**
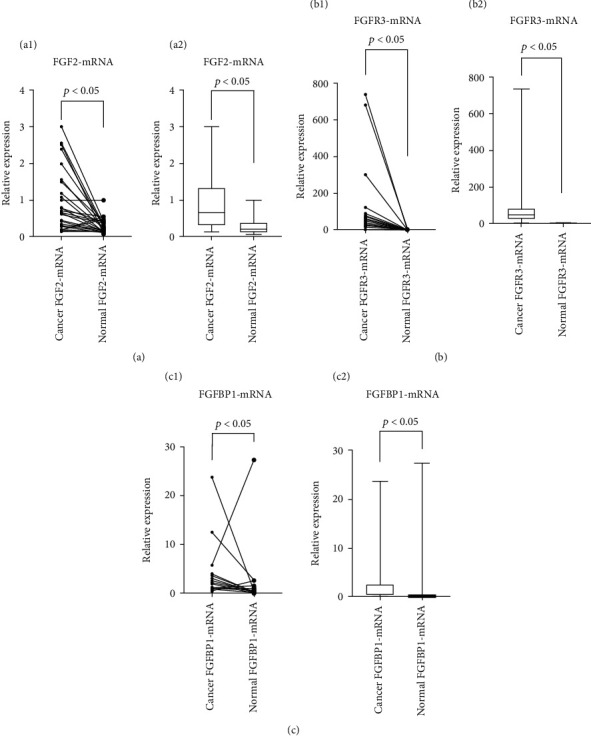
The expression of FGF2, FGFR3, and FGFBP1-mRNA in cancer tissues was significantly higher than that in adjacent tissues (*p* < 0.05).

The results showed that mRNA expressions of FGF2 (*p* < 0.001), FGFR3 (*p* < 0.001), and FGFBP1 (*p* = 0.001) were higher in cancer tissues than in the adjacent tissues (*p* < 0.05) (Figure 2). The difference was statistically significant. The results were consistent with the immunohistochemical results.

### 3.4. Prognostic Factors for OS and PFS

**Figure 3:**
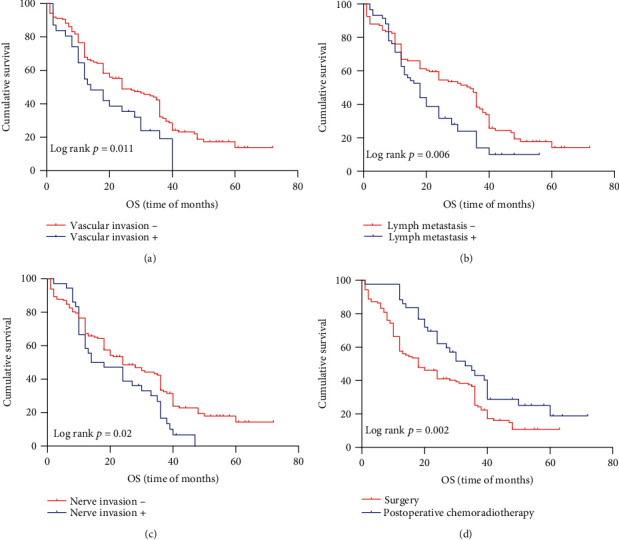
Kaplan–Meier curves for overall survival of ESCC with lymph node metastasis, vascular invasion, nerve invasion, and the treatment of surgery plus chemotherapy. (a) Patients with vascular invasion have a significantly shorter survival (*p* = 0.011); (b) patients with lymph node metastasis have a significantly shorter survival (*p* = 0.006); (c) patients with nerve invasion have a significantly shorter survival (*p* = 0.02); (d) patients who had postoperative chemoradiotherapy have a significantly longer survival (*p* = 0.002).

**Figure 4:**
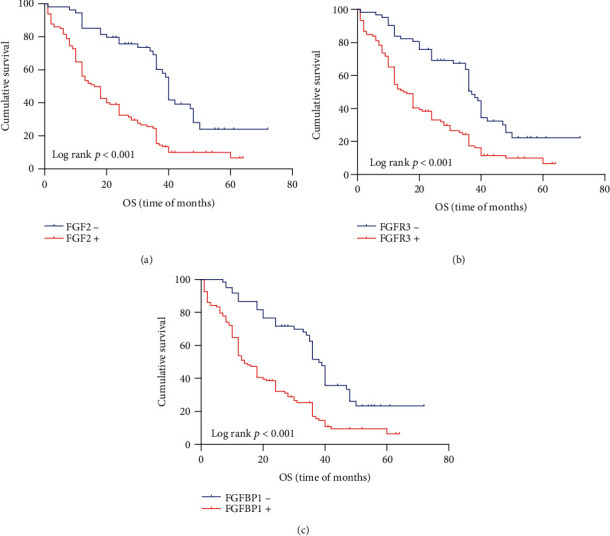
Kaplan–Meier curves for overall survival of ESCC with FGF2, FGFR3, and FGFBP1 expression. (a) Patients expressing high level of FGF2 have a significantly shorter survival (*p* < 0.001); (b) patients expressing high level of FGFR3 have a significantly shorter survival (*p* < 0.001); (c) patients expressing high level of FGFBP1 have a significantly shorter survival (*p* < 0.001).

**Figure 5:**
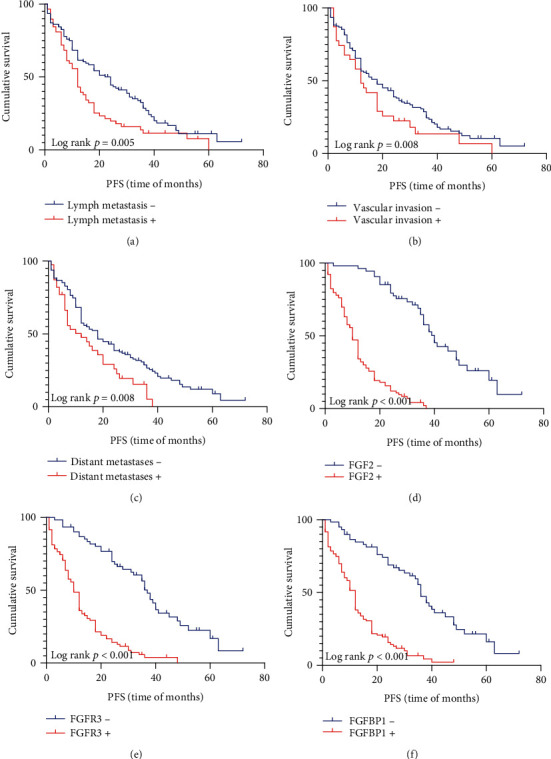
Kaplan–Meier curves for PFS of ESCC. (a) Patients with lymph node metastasis have a significantly shorter survival (*p* = 0.005); (b) patients with vascular invasion have a significantly shorter survival (*p* = 0.008); (c) the prognosis of patients with distant metastasis is poor (*p* = 0.008); (d) patients expressing high level of FGF2 have a significantly shorter survival (*p* < 0.001); (e) patients expressing high level of FGFR3 have a significantly shorter survival (*p* < 0.001); (f) patients expressing high level of FGFBP1 have a significantly shorter survival (*p* < 0.001).

Kaplan–Meier method was used to investigate the relationship between protein expression level and survival rate. Four were lost to follow-up in the 172 patients. Through K–M single-factor analysis, the overall survival rate was closely correlated with FGF2 (*p* < 0.001), FGFBP1 (*p* < 0.001), FGFR3 expression (*p* < 0.001), lymph node metastasis (*p* = 0.006), vascular invasion (*p* = 0.011), nerve invasion (*p* = 0.02), and the treatment with postoperative chemoradiotherapy (*p* = 0.002) (Figures 3 and 4). However, there was no obvious relation, with sex, tumor size, degree of differentiation, and TNM. And progression-free survival is closely related to FGF2 (*p* < 0.001), FGFR3 (*p* < 0.001), and FGFBP1 (*p* < 0.001); lymph node metastasis (*p* = 0.005); vascular invasion (*p* = 0.008), and distant metastases (*p* = 0.008), as is shown in Figure 5 and Table 6. The progression-free survival of FGF2-, FGFR3-, and FGFBP1-positive patients was significantly lower than that of negative patients. Cox multivariate regression analysis showed that, as is shown in Table 6, vascular invasion (*p* = 0.03) and postoperative chemotherapy (*p* = 0.001) can significantly have an impact on the overall survival status of ESCC patients (Table 6). FGF2 (*p* < 0.001), FGFR3 (*p* = 0.003), vascular invasion (*p* = 0.033) and distant metastases (*p* = 0.014) can significantly have an influence on the progression-free survival status of patients (Table 6).

To sum up, we analyze the impact of various factors on the overall survival and progression-free survival. Patients with vascular metastases have a worse prognosis. Postoperative chemotherapy patients have a better prognosis than those who only underwent surgery. Patients with distant metastasis have a worse prognosis. The survival time of patients with lymph node metastasis was significantly lower than that of patients without lymph node metastasis. The overall survival of FGF2-, FGFR3-, and FGFBP1-positive patients is significantly lower than that of negative patients. For the patients with low expression of FGF2, FGFR3, and FGFBP1 are longer than that of patients with high expression in progression-free survival.

### 3.5. The Expression of FGF2, FGFR3, FGFBP1 Effect Overall Survival and Progression-Free Survival Time in Patients with Postoperative Chemotherapy

**Figure 6:**
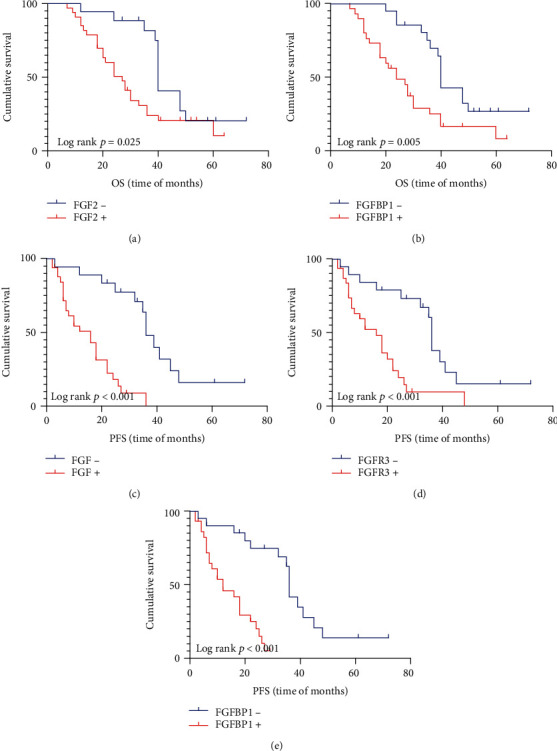
Kaplan–Meier survival analysis: total and progression-free survival in patients undergoing postoperative chemotherapy. (a) The OS of patients with FGF2 overexpression was shorter in ESCC (*p* = 0.025); (b) the high expression of FGFR3 had a poor OS in ESCC (*p* = 0.05); (c) the high expression of FGFBP1 had a poor OS in ESCC (*p* = 0.005); (c) the PFS of patients with FGF2 overexpression was shorter in ESCC (*p* < 0.001); (e) the high expression of FGFR3 had a poor PFS in ESCC (*p* < 0.001); (f) the high expression of FGFBP1 had a poor PFS in ESCC (*p* < 0.001).

In our study, there are 51 patients (30.3%) underwent postoperative chemotherapy. 33 (64.7%) cases had high expression of FGF2, and 18 (35.3%) cases had negative expression. There were 32 positive cases of FGFR3 and 19 negative cases, with a positive rate of 62.7%. There were 30 cases (58.8%) who had high expression of FGFBP1 and 21 (41.2%) cases who had negative expression, as is shown in Table 7 and Figure 6. The results showed that patients with ESCC receiving postoperative chemoradiotherapy had longer survival time, but these patients with high expression of FGF2 (*p* = 0.05), FGFR3 (*p* = 0.025), and FGFBP1 (*p* = 0.005) had significantly lower overall survival than those with negative expression. In progression-free survival, patients with high expression of FGF2 (*p* < 0.001), FGFR3 (*p* < 0.001), and FGFBP1 (*p* < 0.001) had significantly lower survival than those with low expression.

## 4. Discussion

Fibroblast growth factor (FGF) represents 20 different proteins that are widely expressed in various tissues. Fibroblast growth factor (FGF) is involved in biological development and tissue homeostasis maintenance and is associated with angiogenesis and cancer progression [25]. FGF2 (basic FGF) is one of the most thoroughly studied members of the FGF family and has been shown to play a variety of biological roles in different cell and organ systems. It has also been shown to be involved in tumorigenesis and angiogenesis [13]. FGF2 in the tumor microenvironment plays a key role in regulating cancer stem-like cells (CSCs) [26], promoting the occurrence and development of tumors. In fibrosarcoma, cancer cells and their surrounding immune inflammatory cells overexpress or induce FGF2 expression, which plays a key role in tumor progression and angiogenesis [27]. In breast cancer cells, FGF2 showed high expression and was a powerful mitogen and an effective antiapoptotic substance, while inducing its invasion [28, 29]. Takase et al. analyzed the tissue specimens of 70 cases of esophageal squamous cell carcinoma by immunohistochemistry to investigate whether the expression level of FGF2 is related to the clinicopathological parameters of ESCC patients. According to the expression level of FGF2, they were further classified into weak positive group and strong positive group. The results showed that the strong positive group was positively correlated with the depth of infiltration, degree of vascular infiltration, and stage [30]. The results of our study showed that the expression of ESCC patients was significantly higher than that of normal esophageal tissues, and the positive rate was 68% (117/172), which was consistent with previous studies. Moreover, the expression of FGF2-mRNA in the cancer tissues was significantly higher than that in the adjacent tissues in RT-PCR test, which was consistent with the results of IHC. The high expression of FGF2 was related to the tumor size (*p* = 0.026), gender (*p* = 0.047), and lymph metastasis (*p* = 0.007), while the expression of FGF2 was not correlated with the race, age, tumor site, and pathological stage of ESCC cases and so on (*p* > 0.05). The expression of FGF2 is related to gender and may be related to the male-to-female ratio of patients. Therefore, it can be speculated that FGF2 may be a promoting effect on the tumor progression. After survival analysis, the prognosis of FGF2 positive was significantly worse. FGF2 expression is considered an independent prognostic factor affecting the progression-free survival (*p* < 0.001) ESCC patients by Cox multivariate regression analysis. These results suggest that FGF2 may be used as an independent prognostic indicator of progression-free survival in patients with esophageal squamous cell carcinoma.

FGFR3 germ line mutations cause fatal dysplasia, cartilage growth not congruent, and congenital disorders. FGFR3 somatic mutations or excessive FGFR3 protein expression can lead to the development of a variety of malignant tumors. FGFR3 excessive gene mutation and protein expression were first discovered in bladder tumor. Besides, FGFR3 overexpression was found in gastric cancer and liver cancer. Studies have shown that dysfunction in FGFR3 or mutations of FGFR3 are highly associated with multiple cancers, such as multiple myeloma, bladder cancer [31], breast cancer [32], and colorectal cancer [33]. The activation of the FGFR3 signaling pathway can promote tumor growth, metastasis, and drug resistance [34, 35]. Studies have shown that that FGFR3 expression promoted tumor cell proliferation immunohistochemical analysis of early esophageal squamous cell carcinoma [36]. In previous studies of esophageal cancer, FGFR3 was associated with tumor proliferation. In this study, the expression of FGFR3 in ESCC patients was significantly higher than that in tissues adjacent to carcinoma (64.5% (111/172)), which was consistent with FGFR3 expression in gastric and liver cancers in previous studies. And in RT-PCR, FGFR3-mRNA expression in cancer tissues was significantly higher than that in adjacent tissues (*p* < 0.001). Furthermore, the expression of FGFR3 in ESCC carcinoma was significantly higher than that in surrounding normal tissues. In this study, FGFR3 expression was associated with tumor differentiation (*p* = 0.043 and *p* < 0.05), lymph node metastasis (*p* = 0.078 and *p* < 0.1), and race (*p* = 0.033 and *p* < 0.05), suggesting that FGFR3 may have an influence on the tumor development. FGFR3 expression is considered an independent prognostic factor affecting the overall survival (*p* < 0.001) and the progression-free survival (*p* = 0.003) in patients with ESCC by Cox multivariate regression analysis. The survival analysis showed that the FGFR3-positive patients had a poor prognosis. In this study, only advanced esophageal squamous cell carcinoma was selected, because of the greater influence of early and advanced cancers on the prognosis. The previous studies have suggested that FGFR3 may be of diagnostic value in early carcinoma, and further study of FGFR3 expression in early carcinoma of esophageal squamous cell carcinoma is warranted.

In the normal adult tissues, some studies have shown that FGFBP1 has also been shown to induce tumorigenic potential in epithelial cells [37] and to be highly expressed in oral cancer cell lines and tissues [38]. And other studies have found that overexpression of FGFBP1 can lead to skin diseases, such as psoriasis, actinic keratosis, and squamous cell carcinoma of the skin [39]. The previous studies have found that ed-71 (an anticancer agent for squamous cell carcinoma) inhibits tumor growth by inhibiting tumor angiogenesis in squamous cell carcinoma of the skin. One of the functions of ed-71 is to regulate the expression of HBp17/fgfbp-1 in tumors, which can affect the release of fgf-2 by ECM and angiogenesis [40, 41]. However, the expression in ESCC is unclear. The expression of this research shows that FGFBP1 from patients with esophageal is a high expression (70.2%), thus making the results consistent with the expression of colon cancer, pancreatic cancer, and breast cancer. Moreover, RT-PCR further confirmed that the expression of FGFBP1 in ESCC tissues was higher than that in normal tissues (*p* = 0.001). The expression of FGFBP1 with ESCC cases of tumor differentiation (*p* = 0.012), age (*p* = 0.045), and lymph node metastasis (*p* = 0.032) has more obvious relationship, rather than gender, tumor size, tumor location, pathological stage, and vascular invasion of clinical pathology features. We can speculate that FGFBP1 may promote the development of esophageal squamous cell carcinoma through FGF2. Through survival analysis, there was a significant difference in survival time between FGFBP1-positive patients and negative patients. Patients with high expression of FGFBP1 had worse prognosis.

**Figure 7:**
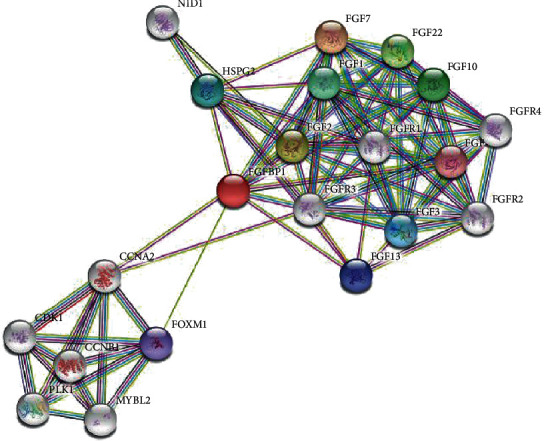
The connection diagram of FGF2, FGFR3, and FGFBP1.

In the previous studies, the FGF/FGFR system is a key factor in tumor-microenvironment interactions [42, 43]. We found that fibroblast growth factor-binding protein (FGFBP1) was the carrier molecule of FGF2, which was first found in tumor cell lines [44]. Subsequent studies have shown that FGFBP1 binds to the FGF2 released from HSPG and then transports the bound FGF2 to the target cell surface [45, 46] to bind to FGFR. FGF/FGFR3 axis may induce carcinogenic effects by promoting cancer progression and increasing angiogenesis potential, leading to metastatic tumor phenotypes (Figure 7). Blocking one or more components of the FGFR signal pathway is being examined in preclinical studies and some clinical trials. However, early findings revealed that alterations in the FGFR gene do not occur uniformly across the various types/subtypes of cancer, suggesting the existence of complex interactions that vary between cancer types/subtypes [47, 48]. The results of our study showed a positive correlation among these factors, suggesting that the FGF2-FGFR3 axis may play a certain role in promoting the occurrence of ESCC and affecting the prognosis of ESCC.

The studies have shown that FGF2 is frequently dysregulated in cancer, especially in advanced stages of disease. The upregulation of FGF2 or FGFRs can promote resistance to chemotherapy. FGF2 is currently being evaluated in clinical studies as a potential predictive biomarker for hematological and solid tumors. FGF2/FGFR inhibitors are being developed and evaluated as monotherapy or as part of a combination therapy for the treatment of different types of cancer [49]. The finding was also found in our study that the survival times of patients with negative expression of FGF2, FGFR3, and FGFBP1 were significantly higher than that of patients with positive expression in 51 patients who received postoperative chemotherapy. It is concluded that the high expression of FGF2, FGFR3, and FGFBP1 in patients with ESCC may be prone to be resistant to chemotherapeutic drugs or radiotherapy. This is consistent with the relevant reports. Therefore, the inhibition of FGF2, FGFR3, and FGFBP1 may enhance the efficacy of chemotherapy, which is hopeful to make it an irreplaceable sensitizing target for cancer treatment. In this study, only immunohistochemistry and PCR were used. Further verification should be done by cell or animal tests.

In summary, the high expression of FGF2 was related to the tumor size of ESCC tissues and lymph node metastasis; the FGFR3 expression was associated with tumor differentiation, race, and lymph node metastasis. The expression of FGFBP1 with ESCC was associated with tumor differentiation degree, age, and lymph node metastasis. The protein and mRNA expressions of FGF2, FGFR3, and FGFBP1 were higher in the ESCC than in the adjacent tissues. FGF2, FGFBP1, and FGFR3 can promote the ESCC progression. FGF2 was significantly correlated with FGFR3 and FGFBP1, and FGFR3 was correlated with FGFBP1. The study further confirmed that the FGF2-FGFR3 axis may promote the progression of esophageal squamous cell carcinoma. The FGF2-FGFR3 axis may be a new direction of targeted therapy for esophageal squamous cell carcinoma. Furthermore, high expression of FGF2, FGFR3, and FGFBP1 may increase drug resistance and reduce survival. Therefore, blocking the FGF2-FGFR3 axis may inhibit the development of tumors. The inhibition of FGF2, FGFR3, and FGFBP1 may be further increased susceptibility to other chemotherapy drugs. The results of multivariate analysis showed that both FGF2 and FGFR3 affected prognosis. Therefore, FGF2 and FGFR3 may be used as molecular markers for prognosis of ESCC.

## Figures and Tables

**Table 1:** Clinicopathological characteristics of esophageal squamous cell carcinoma patients (*n* (%)).

Characteristics and finding	*n* = 172
Age (years old)	63.53 (38-83)
Tumor size (cm)	3.81 (1-8.5)
Gender	
Male	129 (75)
Female	43 (25)
Race	
Han	94 (54.7)
Kazak	78 (45.3)
Tumor site	
Upper	8 (4.7)
Middle	95 (55.2)
Lower	69 (40.1)
Differentiation	
Well	33 (19.2)
Moderate	97 (56.4)
Poor	42 (24.4)
pTNM	
IB	12 (7)
IIA,B	86 (50)
IIIA,B,C	74 (43)
Lymph metastasis	
Negative	113 (65.7)
Positive	59 (34.3)
Vessel invasion	
Negative	141 (82)
Positive	31 (18)
Nerve invasion	
Negative	136 (79.1)
Positive	36 (20.9)
Distant metastases	
Negative	132 (76.7)
Positive	40 (23.3)
Treatment	
Surgery	109 (63.4)
Postoperative chemoradiotherapy	63 (36.6)
FGF2	
Low expression	55 (32)
High expression	117 (68)
FGFR3	
Low expression	61 (35.5)
High expression	111 (64.5)
FGFBP1	
Low expression	60 (34.9)
High expression	112 (65.1)

**Table 2:** PCR primer sequences.

Gene name		Sequence
FGF2	Forward	TTCAAGCAGAAGAGAGAGGAG
	Reverse	TCCGTAACACATTTAGAAGCC

FGFR3	Forward	ACCAAGCCTGTCACCGTAG
	Reverse	CAGAAACTCCCGCAGGTTACC

FGFBP1	Forward	GGGAGGAGCTGTGAGTAACG
	Reverse	CAGGCAGTGCGAGTGAATTG

**Table 3:** Relationship between expression of FGF2, FGFR3, and FGFBP1 in ESCC and clinicopathological parameters (*n*).

Characteristic	Total	FGF2 expression *n*		*p*	FGFR3 expression *n*		*p*	FGFBP1 expression *n*		*p*
	*n* = 172	Negative	Positive		Negative	Positive		Negative	Positive	
Gender				0.047			0.519			0.712
Male	129	36	93		44	85		46	83	
Female	43	19	24		17	26		14	29	
Race				0.794			0.033			0.302
Han	94	31	63		40	54		24	54	
Hazak	78	24	54		21	57		36	58	
Age (years)				0.053			0.384			0.045
<65	91	35	56		35	56		38	53	
≥65	81	20	61		26	55		22	59	
Tumor size				0.026			0.885			0.811
<3 cm	44	20	24		16	28		16	28	
≥ 3 cm	128	35	93		45	83		44	84	
Tumor site				0.902			0.548			0.457
Upper	8	3	5		4	4		4	4	
Middle	95	31	64		35	60		35	60	
Lower	69	21	48		22	47		21	48	
Differentiation				0.231			0.043			0.012
Well	33	11	22		12	21		15	18	
Moderate	97	35	62		43	54		41	56	
Poor	42	9	33		6	36		4	38	
Pathological stage				0.325			0.897			0.320
IB	12	6	6		5	7		6	6	
IIA,B	86	28	58		30	56		32	54	
IIIA,B,C	74	21	53		26	48		22	52	
Lymph metastasis				0.007			0.078			0.032
Negative	113	44	69		45	68		45	68	
Positive	59	11	48		16	43		15	48	
Vascular invasion				0.644			0.68			0.735
Negative	141	44	97		51	90		50	91	
Positive	31	11	20		10	21		10	21	
Nerve invasion				0.313			0.764			0.826
Negative	136	46	90		49	87		48	88	
Positive	36	9	27		12	24		12	24	
Distant metastases				0.142			0.494			0.718
Negative	132	46	86		45	87		47	85	
Positive	40	9	31		16	24		13	27	

**Table 4:** Correlation between FGF2 and FGFR3 and FGFBP1.

	FGF2		*p*	rs
	Positive	Negative		
FGFR3				
Positive	99 (57.5%)	12 (7%)	<0.001	0.612
Negative	18 (10.5%)	43 (25%)		
FGFBP1				
Positive	101 (58.7%)	11 (6.4%)	<0.001	0.649
Negative	16 (9.3%)	44 (25.6%)		

**Table 5:** Correlation between FGFR3 and FGFBP1.

	FGFR3		*p*	rs
	Positive	Negative		
FGFBP1				
Positive	98 (57%)	14 (8.1%)	<0.001	0.656
Negative	13 (7.6%)	47 (27.3%)		

**Table 6:** Univariable and multivariable analyses for overall survival and progression-free survival.

Variable	Overall survival				Progression-free survival			
	Univariable		Multivariable		Univariable		Multivariable	
	Median	*p* value	HR (95% CI)	*p* value	Median	*p* value	HR (95% CI)	*p* value
Gender								
Male	24	0.286			15	0.127		
Female	36				18			
Race								
Han	24	0.604			18	0.515		
Kazakh	24				13			
Age (years)								
<65	28	0.101			18	0.292		
≥65	15				12			
Tumor size								
<3 cm	36	0.203			18	0.839		
≥3 cm	20				15			
Tumor site								
Upper	36	0.31			48	0.178		
Middle	20				12			
Lower	24				18			
Differentiation								
Well	30	0.422			18	0.158		
Moderate	21				14			
Poor	24				20			
pTNM		0.065				0.252		
IB	34				36			
IIA.B	24				16			
IIIA.B,C	20				14			
Lymph metastasis								
Negative	34	0.006	0.75 (0.51-1.09)	0.131	24	0.005	0.912 (0.631-1.319)	0.626
Positive	18				12			
Vascular invasion		0.011	0.60 (0.38-0.95)	0.03		0.008	0.616 (0.394-0.963)	0.033
Negative	24				18			
Positive	14				12			
Nerve invasion								
Negative	24	0.02	0.76 (0.49-1.16)	0.204	15	0.369		
Positive	14				16			
Distant metastases		0.934				0.008	0.597 (0.395-0.903)	0.014
Negative	21				18			
Positive	24				12			
Treatment								
Surgery	15	0.002	0.54(0.36-0.76)	0.001	13	0.259		
Postoperative chemoradiotherapy	33				20			
FGF2								
Negative	40	<0.001	0.57 (0.32-1.01)	0.056	40	<0.001	0.183 (0.097-0.364)	<0.001
Positive	16				10			
FGFR3								
Negative	37	<0.001	0.69 (0.42-1.15)	0.157	37	<0.001	0.465 (0.281-0.769)	0.003
Positive	15				10			
FGFBP1								
Negative	38	<0.001	0.84 (0.48-1.49)	0.541	36	<0.001	0.870 (0.477-1.587)	0.65
Positive	14				12			

**Table 7:** Univariate analysis of overall and progression-free survival in patients with ESCC postoperative chemoradiotherapy.

Variable		Overall survival			Progression-free survival		
		Median	Log rank (chi-square)	*p*	Median	Log rank (chi-square)	*p*
FGFR3	Negative	40	3.691	0.05	36	12.154	<0.001
	Positive	28			16		

FGF2	Negative	40	5.047	0.025	36	20.163	<0.001
	Positive	27			16		

FGFBP1	Negative	40	7.901	0.005	36	20.403	<0.001
	Positive	24			12		

## Data Availability

The data of immunohistochemical and PCR used to support the findings of this study are included within the supplementary information file(s). The data used to support the findings of this study were supplied by Yuqing Ma under license. Because it involves patient privacy, so cannot be made freely available. Requests for access to these data should be made to Yuqing Ma (yuqingm0928@126.com).

## Supplementary Materials

Supplementary materials Table 1(s)-Table 3(s): the data of immunohistochemical patients. Table 4 s-7 s: mPCR data information.
