# Supplementary material for: The Expression and Prognostic Value of FGF2, FGFR3, and FGFBP1 in Esophageal Squamous Cell Carcinoma
Source: Anal Cell Pathol (Amst). 2020 Dec 11;2020:2872479. doi: 10.1155/2020/2872479 (PMC7748917; doi:10.1155/2020/2872479)
Supplement: Supplementary materials — Table 1(s)-Table 3(s): the data of immunohistochemical patients. Table 4 s-7 s: mPCR data information. [file 2872479.f1.zip › Table 1s.docx]

| Table 1s Immunohistochemical statistics | | | | | | | | | | | | | | | | | | | | | | |
| --- | --- | --- | --- | --- | --- | --- | --- | --- | --- | --- | --- | --- | --- | --- | --- | --- | --- | --- | --- | --- | --- | --- |
| Name | Race | Age (years) | Sex | Tumor site | Tumor size | specimen type | Differentiation | Infiltrating depth | TNM（The eighth edition） | AJCC | Lymph metastasis | Lymph metastasis | Vascular invasion | Vascular invasion | 神经侵犯 | 神经侵犯 | 0S(最新） | PFS | 生存状态 | FGF2 | FGFR3 | FGFBP1 |
| 胡吐拜斯德克 | hazak | 56 | male | Lower | 7 | Uplift type | Poor | Muscular layer | IIIA | 2 | positive | 1 | negative | 0 | negative | 0 | 9 | 9 | 1 | + | + | + |
| 亭努尔汗.哈那皮亚 | hazak | 52 | female | Middle | 2.6 | Ulcer type | Moderate | Muscular layer | ⅡB | 1 | negative | 0 | negative | 0 | positive | 1 | 18 | 9 | 1 | + | + | + |
| 托呼塔尔汗.夏依切马尔坦 | hazak | 61 | male | Lower | 3.2 | Ulcer type | Poor | The outer membrane | ⅢB | 2 | positive | 1 | positive | 1 | positive | 1 | 24 | 18 | 1 | + | + | + |
| 热苏力.哈力木 | hazak | 51 | male | Middle | 4 | Uplift type | Poor | The outer membrane | ⅢB | 2 | positive | 1 | positive | 1 | negative | 0 | 18 | 10 | 1 | + | + | + |
| 叶尔波木森.阿力亚古巴尔 | hazak | 50 | male | Middle | 2.2 | Ulcer type | Moderate | The outer membrane | IIIA | 2 | positive | 1 | positive | 1 | positive | 1 | 10 | 10 | 1 | - | - | - |
| 布看。艾尼 | hazak | 44 | male | Lower | 7.2 | Mushroom umbrella | Poor | The outer membrane | ⅢA | 2 | positive | 1 | positive | 1 | negative | 0 | 28 | 18 | 1 | + | + | + |
| 迪汗别克.萨哈巴 | hazak | 56 | male | Lower | 4 | Ulcer type | Poor | The outer membrane | ⅡA | 1 | negative | 0 | negative | 0 | negative | 0 | 60 | 24 | 1 | + | + | + |
| 木买尔 | hazak | 39 | male | Lower | 5.5 | Ulcer type | well | The outer membrane | ⅠB | 0 | negative | 0 | negative | 0 | negative | 0 | 24 | 22 | 1 | - | - | - |
| 库兰.察依扎 | hazak | 60 | female | Lower | 2.1 | Ulcer type | Moderate | Muscular layer | ⅡA | 1 | negative | 0 | positive | 1 | negative | 0 | 40 | 32 | 1 | - | - | - |
| 叶留.巴合达提 | hazak | 45 | male | Lower | 3.5 | Ulcer type | Moderate | The outer membrane | ⅡA | 1 | negative | 0 | positive | 1 | positive | 1 | 36 | 18 | 1 | + | + | + |
| 西那尔别克·木哈买提哈力 | hazak | 67 | male | Lower | 5.5 | Mushroom umbrella | well | The outer membrane | ⅢA | 2 | positive | 1 | negative | 0 | positive | 1 | 30 | 16 | 1 | + | + | + |
| 胡瓦尼什 | hazak | 50 | male | Middle | 5 | Ulcer type | Moderate | Muscular layer | ⅢA | 2 | positive | 1 | negative | 0 | negative | 0 | 20 | 10 | 1 | + | + | + |
| 库木斯古丽 | hazak | 51 | female | Middle | 5 | Ulcer type | Moderate | Muscular layer | ⅡB | 1 | negative | 0 | negative | 0 | positive | 1 | 10 | 3 | 1 | + | + | + |
| 阿不都拉生.巴拉拜 | hazak | 69 | male | Lower | 3.4 | Uplift type | Moderate | Muscular layer | ⅡA | 1 | negative | 0 | negative | 0 | negative | 0 | 1 | 1 | 1 | + | + | + |
| 阔克西·吐海 | hazak | 66 | male | Lower | 4.3 | Ulcer type | Moderate | The outer membrane | ⅢB | 2 | positive | 1 | positive | 1 | positive | 1 | 8 | 8 | 1 | + | + | + |
| 哈再孜 | hazak | 44 | male | Lower | 2.9 | Ulcer type | well | Muscular layer | ⅠB | 0 | negative | 0 | negative | 0 | negative | 0 | 18 | 15 | 1 | - | + | - |
| 塔依希别克·尔阿合木 | hazak | 63 | male | Middle | 6 | Uplift type | Moderate | Muscular layer | ⅡB | 1 | negative | 0 | positive | 1 | negative | 0 | 6 | 6 | 1 | + | + | + |
| 波开·马克扎木 | hazak | 51 | male | Middle | 6.4 | Ulcer type | Moderate | Muscular layer | IIIA | 2 | positive | 1 | negative | 0 | negative | 0 | 24 | 6 | 1 | + | + | + |
| 阿扎提·卡子 | hazak | 57 | male | Middle | 4 | Uplift type | well | Muscular layer | ⅢA | 2 | positive | 1 | negative | 0 | negative | 0 | 8 | 6 | 1 | + | + | + |
| 阿吾力汗·达开 | hazak | 59 | male | Middle | 7.5 | Uplift type | Moderate | The outer membrane | ⅡB | 1 | negative | 0 | negative | 0 | negative | 0 | 12 | 12 | 1 | - | - | - |
| 吐尔逊 | hazak | 66 | male | Lower | 3.5 | Ulcer type | Poor | Muscular layer | ⅢA | 2 | positive | 1 | negative | 0 | negative | 0 | 40 | 12 | 1 | + | - | - |
| 加孜依拉 | hazak | 38 | female | Lower | 3.5 | Uplift type | Moderate | Muscular layer | ⅡA | 1 | negative | 0 | negative | 0 | negative | 0 | 12 | 1 | 1 | + | + | + |
| 刘荷青 | han | 56 | female | Lower | 2 | Ulcer type | Moderate | Muscular layer | ⅡA | 1 | negative | 0 | positive | 1 | negative | 0 | 30 | 30 | 1 | - | + | - |
| 潘金玲 | han | 58 | male | Lower | 2.8 | Mushroom umbrella | Poor | Muscular layer | ⅡA | 1 | negative | 0 | negative | 0 | negative | 0 | 36 | 18 | 1 | + | - | + |
| 连成玺 | han | 73 | male | Middle | 4 | Uplift type | well | The outer membrane | IIIB | 2 | positive | 1 | negative | 0 | negative | 0 | 12 | 12 | 1 | + | + | + |
| 杨泗成 | han | 79 | male | Middle | 4.5 | Ulcer type | Moderate | The outer membrane | ⅢA | 2 | positive | 1 | negative | 0 | negative | 0 | 36 | 28 | 1 | + | - | - |
| 黄成明 | han | 56 | male | Middle | 2.5 | Ulcer type | Moderate | The outer membrane | ⅡB | 1 | negative | 0 | negative | 0 | negative | 0 | 24 | 10 | 1 | + | + | - |
| 加尼木汉 | hazak | 51 | male | Lower | 2.5 | Uplift type | Poor | The outer membrane | IIIA | 2 | positive | 1 | positive | 1 | negative | 0 | 18 | 6 | 1 | + | + | + |
| 马心芳 | han | 66 | female | Middle | 3 | Ulcer type | Moderate | Muscular layer | ⅡB | 1 | negative | 0 | negative | 0 | negative | 0 | 36 | 24 | 1 | - | - | - |
| 李忠义 | han | 72 | male | Lower | 5 | Uplift type | Moderate | Muscular layer | ⅡB | 1 | positive | 1 | negative | 0 | negative | 0 | 24 | 15 | 1 | + | - | + |
| 高大安 | han | 70 | male | Middle | 5 | Mushroom umbrella | Moderate | The outer membrane | ⅢB | 2 | positive | 1 | negative | 0 | negative | 0 | 24 | 12 | 1 | + | + | + |
| 白坎 | hazak | 67 | female | Lower | 3 | Uplift type | well | Muscular layer | ⅠB | 0 | negative | 0 | negative | 0 | negative | 0 | 6 | 6 | 1 | + | + | + |
| 杨连芳 | han | 57 | female | Lower | 3.5 | Ulcer type | Moderate | Muscular layer | ⅡA | 1 | negative | 0 | negative | 0 | negative | 0 | 10 | 10 | 1 | + | + | + |
| 薛风歌 | han | 63 | male | Middle | 2.8 | Uplift type | Moderate | The outer membrane | ⅢB | 2 | positive | 1 | negative | 0 | negative | 0 | 6 | 6 | 1 | + | - | + |
| 赵月英 | han | 68 | female | Lower | 2.8 | Mushroom umbrella | Moderate | Muscular layer | ⅡA | 1 | negative | 0 | negative | 0 | negative | 0 | 42 | 40 | 1 | - | - | + |
| 赵士学 | han | 69 | male | Middle | 2.4 | Ulcer type | Poor | The outer membrane | IIIA | 2 | negative | 0 | negative | 0 | positive | 1 | 24 | 24 | 1 | + | + | + |
| 宋汉林 | han | 76 | male | Lower | 4 | Medullary type | Poor | The outer membrane | ⅡA | 1 | negative | 0 | negative | 0 | positive | 1 | 10 | 10 | 1 | + | - | - |
| 叶斯布拉提·艾力 | hazak | 63 | male | Lower | 5.2 | Mushroom umbrella | Moderate | Muscular layer | ⅡA | 1 | negative | 0 | negative | 0 | positive | 1 | 6 | 1 | 1 | + | + | + |
| 王秀芳 | han | 65 | female | Lower | 3.5 | Ulcer type | Moderate | The outer membrane | IIIB | 2 | positive | 1 | positive | 1 | positive | 1 | 12 | 12 | 1 | + | - | + |
| 王宝仓 | han | 52 | male | Lower | 4.1 | Ulcer type | Moderate | The outer membrane | ⅢA | 2 | positive | 1 | negative | 0 | negative | 0 | 20 | 6 | 1 | + | - | - |
| 霍军 | han | 78 | male | Lower | 6.3 | Ulcer type | Moderate | The outer membrane | ⅢA | 2 | positive | 1 | positive | 1 | negative | 0 | 2 | 2 | 1 | + | + | + |
| 努尔孜亚·阿布勒哈帕 | hazak | 64 | female | Middle | 2.5 | Uplift type | Moderate | Muscular layer | ⅡB | 1 | negative | 0 | negative | 0 | negative | 0 | 1 | 1 | 1 | - | + | + |
| 万义年 | han | 67 | male | Middle | 1.2 | Uplift type | well | Muscular layer | ⅡA | 1 | negative | 0 | negative | 0 | negative | 0 | 20 | 6 | 1 | + | - | - |
| 黄东升 | han | 55 | male | Lower段 | 3.2 | Ulcer type | Moderate | 全层 | IIIA | 2 | positive | 1 | positive | 1 | negative | 0 | 26 | 26 | 0 | - | - | - |
| 葛兴明 | han | 66 | male | Middle | 4 | Ulcer type | Moderate | Muscular layer | ⅡB | 1 | negative | 0 | negative | 0 | negative | 0 | 2 | 2 | 1 | + | + | + |
| 哈木拉提·热木扎 | hazak | 55 | male | Lower | 3 | Ulcer type | Moderate | The outer membrane | ⅢB | 2 | positive | 1 | negative | 0 | negative | 0 | 18 | 18 | 1 | + | + | + |
| 陈克美 | han | 59 | male | Middle | 3.5 | Ulcer type | Poor | The outer membrane | ⅡB | 1 | negative | 0 | negative | 0 | positive | 1 | 24 | 16 | 1 | + | - | - |
| 艾尼瓦尔别克·居马别 | hazak | 72 | male | Lower | 3 | Ulcer type | Moderate | The outer membrane | ⅡA | 1 | negative | 0 | positive | 1 | negative | 0 | 40 | 6 | 1 | + | + | + |
| 艾扎提别克·队山 | hazak | 50 | male | Lower | 4.5 | Ulcer type | Moderate | The outer membrane | ⅡA | 1 | negative | 0 | negative | 0 | positive | 1 | 33 | 22 | 1 | + | + | - |
