# Supplementary material for: The Expression and Prognostic Value of FGF2, FGFR3, and FGFBP1 in Esophageal Squamous Cell Carcinoma
Source: Anal Cell Pathol (Amst). 2020 Dec 11;2020:2872479. doi: 10.1155/2020/2872479 (PMC7748917; doi:10.1155/2020/2872479)
Supplement: Supplementary materials — Table 1(s)-Table 3(s): the data of immunohistochemical patients. Table 4 s-7 s: mPCR data information. [file 2872479.f1.zip › Table 3s.docx]

| Table 3s Immunohistochemical statistics | | | | | | | | | | | | | | | | | | | | | | |
| --- | --- | --- | --- | --- | --- | --- | --- | --- | --- | --- | --- | --- | --- | --- | --- | --- | --- | --- | --- | --- | --- | --- |
| Name | Race | Age (years) | Sex | Tumor site | Tumor size | specimen type | Differentiation | Infiltrating depth | TNM（The eighth edition） | AJCC | Lymph metastasis | Lymph metastasis | Vascular invasion | Vascular invasion | 神经侵犯 | 神经侵犯 | 0S(最新） | PFS | 生存状态 | FGF2 | FGFR3 | FGFBP1 |
| 塞力汗·阿地开 | hazak | 69 | male | Middle | 3.8 | Ulcer type | Moderate | The outer membrane | ⅢB | 2 | positive | 1 | negative | 0 | positive | 1 | 12 | 35 | 1 | - | - | - |
| 吐尔逊别克·斯玛胡勒 | hazak | 49 | male | Middle | 3.5 | Medullary type | Moderate | Muscular layer | ⅡA | 1 | negative | 0 | positive | 1 | positive | 1 | 40 | 48 | 1 | - | + | - |
| 吾坦·卡斯木拜 | hazak | 62 | male | Lower | 2.5 | Medullary type | Moderate | Muscular layer | ⅡB | 1 | positive | 1 | negative | 0 | negative | 0 | 48 | 3 | 0 | + | + | + |
| 王文泰 | han | 73 | male | Lower | 7.6 | Ulcer type | Moderate | Muscular layer | ⅡA | 1 | negative | 0 | negative | 0 | negative | 0 | 24 | 24 | 1 | - | - | + |
| 达力哈·巴开 | hazak | 55 | female | Middle | 6 | Ulcer type | Poor | Muscular layer | ⅡB | 1 | positive | 1 | negative | 0 | negative | 0 | 3 | 1 | 1 | + | + | + |
| 张志强 | han | 64 | male | Upper | 6.5 | Ulcer type | Poor | Muscular layer | ⅡB | 1 | negative | 0 | negative | 0 | negative | 0 | 35 | 24 | 1 | + | - | - |
| 余生荣 | han | 60 | male | Upper | 4 | Ulcer type | Poor | The outer membrane | ⅢA | 2 | positive | 1 | positive | 1 | negative | 0 | 2 | 2 | 1 | + | + | + |
| 李兆成 | han | 64 | male | Middle | 1.5 | Uplift type | Moderate | Muscular layer | ⅡB | 1 | negative | 0 | negative | 0 | negative | 0 | 36 | 26 | 1 | - | - | - |
| 阿勒提 | hazak | 69 | female | Middle | 2.5 | Medullary type | Poor | Muscular layer | ⅡB | 1 | negative | 0 | negative | 0 | negative | 0 | 12 | 12 | 1 | - | + | + |
| 达列力·吾力斯拜 | hazak | 35 | male | Lower | 4.6 | Ulcer type | Poor | The outer membrane | ⅡA | 1 | negative | 0 | negative | 0 | negative | 0 | 18 | 8 | 1 | + | + | - |
| 申作文 | han | 62 | male | Middle | 8 | Medullary type | Moderate | Muscular layer | ⅢA | 2 | positive | 1 | negative | 0 | negative | 0 | 18 | 18 | 1 | + | + | + |
| 侯光辉 | han | 48 | male | Middle | 3. 5 | Ulcer type | Moderate | Muscular layer | ⅡB | 1 | positive | 1 | negative | 0 | negative | 0 | 28 | 8 | 1 | + | + | + |
| 古丽森·沙德胡马尔 | hazak | 66 | female | Middle | 5 | Ulcer type | Poor | The outer membrane | IIIB | 2 | positive | 1 | positive | 1 | positive | 1 | 8 | 8 | 1 | - | - | - |
| 仝义忍 | han | 79 | male | Middle | 5.2 | Ulcer type | well | Muscular layer | IB | 0 | negative | 0 | negative | 0 | negative | 0 | 12 | 7 | 1 | + | + | + |
| 袁超群 | han | 72 | male | Middle Lower段 | 7.5 | Ulcer type | Moderate | 全层 | IB | 2 | negative | 0 | negative | 0 | negative | 0 | 21 | 5 | 1 | + | + | + |
| 赵和生 | han | 72 | male | Middle | 3.5 | Ulcer type | Moderate | The outer membrane | ⅢA | 2 | positive | 1 | positive | 1 | positive | 1 | 10 | 3 | 1 | + | + | + |
| 赵青兰 | han | 69 | female | Lower | 4 | Uplift type | Poor | Muscular layer | ⅡA | 1 | negative | 0 | negative | 0 | negative | 0 | 12 | 2 | 1 | + | + | + |
| 杨山培 | han | 73 | male | Middle | 2.5 | Ulcer type | Poor | The outer membrane | ⅢB | 2 | positive | 1 | negative | 0 | negative | 0 | 42 | 1 | 0 | + | + | + |
| 高亮亮 | han | 54 | male | Lower | 5 | Ulcer type | Moderate | Muscular layer | ⅡA | 1 | negative | 0 | negative | 0 | positive | 1 | 9 | 7 | 1 | + | + | + |
| 候贵卿 | han | 82 | male | Middle | 3.5 | Ulcer type | Moderate | 全层 | IIIA | 2 | positive | 1 | negative | 0 | negative | 0 | 13 | 13 | 1 | + | + | + |
| 空格娜帕尔 | hazak | 60 | female | Middle | 2.2 | Uplift type | Moderate | The outer membrane | ⅡB | 1 | negative | 0 | negative | 0 | positive | 1 | 41 | 10 | 0 | + | + | + |
| 木巴热克·阿艾卜江 | hazak | 65 | female | Middle | 3 | Ulcer type | well | Muscular layer | ⅡA | 1 | negative | 0 | negative | 0 | positive | 1 | 10 | 10 | 1 | + | + | + |
| 开列别克·斯拉木汗 | hazak | 56 | male | Middle | 1.5 | Uplift type | well | 浆膜层 | IIA | 2 | negative | 0 | negative | 0 | negative | 0 | 61 | 61 | 0 | - | - | - |
| 叶尔扎提·朱玛汗 | hazak | 45 | male | Middle | 3.5 | Ulcer type | well | 深Muscular layer | IB | 1 | negative | 0 | positive | 1 | negative | 0 | 10 | 10 | 1 | + | + | + |
| 切布肯·哈斯木汉 | hazak | 69 | male | Middle | 3 | Uplift type | Moderate | Muscular layer | ⅡB | 1 | negative | 0 | negative | 0 | negative | 0 | 39 | 9 | 0 | + | - | - |
| 巴依阿里 | hazak | 52 | male | Middle | 4 | Ulcer type | Poor | The outer membrane | ⅡB | 1 | negative | 0 | negative | 0 | negative | 0 | 35 | 10 | 0 | + | + | + |
| 蒋福瑞 | han | 60 | male | Middle | 4.5 | Ulcer type | Moderate | 全层 | IIB | 2 | negative | 0 | negative | 0 | negative | 0 | 56 | 56 | 0 | - | - | - |
| 许兰芳 | han | 75 | female | Middle | 3.5 | Ulcer type | well | Muscular layer | IIB | 1 | positive | 1 | negative | 0 | negative | 0 | 56 | 56 | 0 | - | - | - |
| 何立荣 | han | 67 | male | Middle | 2.5 | Uplift type | Moderate | 全层 | IIB | 2 | negative | 0 | positive | 1 | positive | 1 | 14 | 14 | 1 | + | - | + |
| 努曼 | hazak | 73 | male | Middle | 1 | Uplift type | Poor | 全层 | IIA | 2 | negative | 0 | negative | 0 | negative | 0 | 31 | 31 | 1 | + | - | + |
| 李书 | han | 75 | male | Middle | 6 | Ulcer type | Moderate | 全层 | IIA | 2 | negative | 0 | negative | 0 | negative | 0 | 1 | 1 | 1 | + | + | + |
| 魏永福 | han | 66 | male | Middle | 2.5 | Uplift type | Poor | 全层 | IIIA | 2 | positive | 1 | negative | 0 | positive | 1 | 29 | 29 | 0 | + | + | + |
| 陈万德 | han | 69 | male | Upper | 5 | Ulcer type | well | 全层 | II | 2 | negative | 0 | negative | 0 | negative | 0 | 14 | 14 | 0 | + | + | + |
| 阿思力旦尼 | hazak | 72 | male | Middle | 3 | Ulcer type | Moderate | The outer membrane层 | IIA | 2 | negative | 0 | negative | 0 | positive | 1 | 27 | 27 | 1 | + | + | + |
| 祁彦俊 | han | 67 | male | Middle | 5 | Ulcer type | well | 全层 | II | 2 | negative | 0 | negative | 0 | negative | 0 | 37 | 37 | 1 | + | - | + |
| 涂修祥 | han | 59 | male | Middle | 3.5 | Ulcer type | Moderate | 全层 | IIB | 2 | negative | 0 | negative | 0 | negative | 0 | 34 | 34 | 1 | - | + | - |
| 杨其超 | han | 68 | male | Middle | 5 | Ulcer type | well | Muscular layer | IB | 0 | negative | 0 | negative | 0 | negative | 0 | 48 | 44 | 0 | - | - | - |
| 唐玉莲 | han | 66 | female | Middle | 3 | Ulcer type | Moderate | 全层 | IIB | 2 | negative | 0 | negative | 0 | negative | 0 | 48 | 48 | 0 | - | - | - |
| 吕生润 | han | 74 | male | Middle | 1 | Uplift type | Moderate | Muscular layer | IIA | 1 | negative | 0 | negative | 0 | negative | 0 | 48 | 48 | 0 | - | - | + |
| 苏兰·提列吾别克 | hazak | 58 | male | Middle | 2.5 | Uplift type | Moderate | The outer membrane层 | IIA | 2 | negative | 0 | negative | 0 | positive | 1 | 39 | 39 | 1 | - | - | - |
| 张三辛 | han | 69 | male | Middle | 3 | Ulcer type | well | Muscular layer | IIB | 1 | negative | 0 | negative | 0 | negative | 0 | 1 | 1 | 1 | + | + | + |
| 周美芳 | han | 66 | female | Middle | 3 | Ulcer type | well | 全层 | IIIA | 2 | positive | 1 | negative | 0 | negative | 0 | 44 | 44 | 0 | - | + | - |
| 杨桂荣 | han | 73 | female | Middle | 4.5 | Ulcer type | Moderate | 全层 | IIIC | 2 | positive | 1 | negative | 0 | negative | 0 | 44 | 38 | 0 | - | - | - |
| 尼斯甫哈力 | hazak | 64 | male | Middle | 3.5 | Ulcer type | Moderate | 深Muscular layer | IIB | 1 | positive | 1 | negative | 0 | positive | 1 | 8 | 8 | 1 | + | + | + |
| 陈荣光 | han | 56 | male | Middle | 4.0 | Ulcer type | Moderate | 全层 | IIIC | 2 | positive | 1 | positive | 1 | negative | 0 | 12 | 12 | 1 | - | + | + |
| 王桂珍 | han | 64 | female | Middle | 2.5 | Uplift type | Moderate | 黏膜层 | IB | 0 | negative | 0 | negative | 0 | negative | 0 | 40 | 40 | 0 | - | - | - |
| 洪乐 | han | 55 | male | Middle | 3.2 | Ulcer type | well | 全层 | IIIA | 2 | negative | 0 | negative | 0 | positive | 1 | 35 | 35 | 1 | - | - | - |
| 安尼瓦尔别克·朱马别克 | hazak | 73 | male | Middle | 2.5 | Uplift type | Moderate | Muscular layer | IIA | 1 | negative | 0 | negative | 0 | negative | 0 | 2 | 2 | 1 | + | + | + |
| 林瑞英 | han | 55 | female | Middle | 4.5 | Ulcer type | Moderate | Muscular layer | IIB | 1 | negative | 0 | negative | 0 | negative | 0 | 36 | 36 | 0 | - | - | - |
| 庞安玉 | han | 78 | male | Middle | 4.5 | Ulcer type | well | Muscular layer | IIA | 1 | positive | 1 | negative | 0 | negative | 0 | 8 | 8 | 1 | + | + | - |
| 杨发香 | han | 65 | female | Middle | 2.5 | Uplift type | Moderate | 全层 | IV | 2 | negative | 0 | negative | 0 | negative | 0 | 12 | 12 | 1 | + | + | + |
| 李建福 | han | 56 | male | Middle | 3.8 | Ulcer type | Moderate | Muscular layer | IIB | 1 | negative | 0 | positive | 1 | positive | 1 | 33 | 33 | 0 | - | - | - |
| 洪瑞林 | han | 74 | male | Middle | 5.0 | Ulcer type | Moderate | 全层 | IIB | 2 | negative | 0 | negative | 0 | negative | 0 | 7 | 7 | 1 | + | + | - |
| 张玉兰 | han | 70 | female | Lower | 2.5 | Uplift type | Moderate | Muscular layer | IV | 1 | positive | 1 | negative | 0 | negative | 0 | 18 | 3 | 0 | + | + | + |
| 沈伯余 | han | 72 | male | Lower | 3.5 | Ulcer type | Moderate | 全层 | IV | 2 | positive | 1 | negative | 0 | positive | 1 | 14 | 7 | 1 | + | + | + |
| 徐银如 | han | 64 | male | Lower | 2.2 | Uplift type | Moderate | 全层 | IIIA | 2 | positive | 1 | negative | 0 | negative | 0 | 7 | 7 | 1 | + | + | + |
| 张家瑞 | han | 61 | male | Middle | 5.5 | Ulcer type | Moderate | Muscular layer | IIB | 2 | negative | 0 | positive | 1 | negative | 0 | 28 | 28 | 0 | - | - | - |
| 景真 | han | 66 | male | Lower | 4 | Ulcer type | Moderate | 全层 | IIIA | 2 | positive | 1 | negative | 0 | negative | 0 | 15 | 15 | 1 | + | + | + |
| 达吾列提汗·阿衣提 | hazak | 63 | male | Lower | 0.6 | Uplift type | well | 中Muscular layer | IIA | 1 | negative | 0 | negative | 0 | negative | 0 | 27 | 27 | 0 | - | - | + |
| 陈仕俊 | han | 70 | male | Upper | 4 | Ulcer type | Moderate | 全层 | IV | 2 | negative | 0 | negative | 0 | negative | 0 | 27 | 27 | 0 | - | - | - |
| 马振荣 | han | 59 | male | Lower | 4 | Ulcer type | Moderate | 全层 | IIIA | 2 | positive | 1 | positive | 1 | positive | 1 | 13 | 13 | 1 | + | + | + |
| 王东斌 | han | 78 | male | Middle | 3.0 | Ulcer type | Poor | 全层 | IIB | 2 | negative | 0 | negative | 0 | negative | 0 | 1 | 1 | 1 | + | + | + |
| 那扎尔别克·亲俄特 | hazak | 54 | male | Middle | 3 | Ulcer type | well | Muscular layer | IB | 0 | negative | 0 | negative | 0 | negative | 0 | 23 | 5 | 0 | + | + | + |
| 江思力汗·热合木 | hazak | 50 | female | Middle | 3.5 | Ulcer type | well | 全层 | IIA | 2 | negative | 0 | negative | 0 | negative | 0 | 23 | 23 | 0 | - | - | + |
| 萨马尔汗·合尔合孜百 | hazak | 67 | male | Middle | 2.5 | Uplift type | Moderate | 全层 | IIA | 2 | negative | 0 | negative | 0 | negative | 0 | 5 | 5 | 1 | + | + | + |
| 白德万·玛合木提 | hazak | 66 | female | Lower | 2 | Uplift type | Moderate | 全层 | IIA | 2 | negative | 0 | negative | 0 | negative | 0 | 22 | 22 | 0 | - | + | + |
| 李凤勇 | han | 49 | male | Middle | 5.5 | Ulcer type | Moderate | 全层 | IV | 2 | positive | 1 | negative | 0 | negative | 0 | 13 | 4 | 1 | + | + | + |
| 顾克义 | han | 74 | male | Middle | 4.5 | Ulcer type | Moderate | 全层 | IIIB | 2 | positive | 1 | negative | 0 | negative | 0 | 7 | 2 | 1 | + | + | + |
| 范怀木 | han | 62 | male | Middle | 4 | Ulcer type | well | 全层 | IV | 2 | negative | 0 | negative | 0 | negative | 0 | 10 | 10 | 1 | + | - | + |
| 巴合提古丽·博勒斯别克 | hazak | 49 | female | Lower | 4 | Ulcer type | well | 深Muscular layer | IIA | 2 | negative | 0 | negative | 0 | negative | 0 | 18 | 18 | 0 | + | + | + |
| 努丽合扎尔 | hazak | 73 | male | Lower | 3 | Ulcer type | Moderate | 全层 | IIA | 1 | negative | 0 | negative | 0 | negative | 0 |  |  |  | - | + | + |
| 切尔克莆 | hazak | 64 | male | Lower | 5 | Ulcer type | well | 深Muscular layer | IIA | 1 | negative | 0 | negative | 0 | negative | 0 |  |  |  | + | + | + |
| 库兰达 沙黑多拉 | hazak | 58 | female | Middle | 5.5 | Ulcer type | well | 深Muscular layer | IIA | 1 | negative | 0 | negative | 0 | negative | 0 |  |  |  | + | + | + |
| 莎黑都拉 | hazak | 76 | male | Lower | 3 | Ulcer type | Moderate | 深Muscular layer | IIA | 1 | negative | 0 | negative | 0 | negative | 0 |  |  |  | + | + | + |
