# Supplementary material for: The Expression and Prognostic Value of FGF2, FGFR3, and FGFBP1 in Esophageal Squamous Cell Carcinoma
Source: Anal Cell Pathol (Amst). 2020 Dec 11;2020:2872479. doi: 10.1155/2020/2872479 (PMC7748917; doi:10.1155/2020/2872479)
Supplement: Supplementary materials — Table 1(s)-Table 3(s): the data of immunohistochemical patients. Table 4 s-7 s: mPCR data information. [file 2872479.f1.zip › Table 4s FGF2.docx]

| Table 4s. FGF2-mRNA | | | | | | | | | | | | | | | | |
| --- | --- | --- | --- | --- | --- | --- | --- | --- | --- | --- | --- | --- | --- | --- | --- | --- |
| sample | Primer-FGF2 | Cancer-CT mean | actin | CT mean | CT FGF2-CTactin | 2-ΔCT | ΔΔCT | 2-ΔΔCT | sample | primer-FGF2 | Normal-CT mean | actin | CT mean | CT FGF2-CTactin | ΔΔCT | 2-ΔΔCT |
| 1C | 26.601 | 27.0396667 | 17.557 | 17.7323333 | 9.282 | 0.00160635 | 1 | 1 | 1L | 27.107 | 27.128 | 20.653 | 20.6966667 | 6.432 | 1 | 1 |
|  | 27.996 |  | 17.689 |  |  |  |  |  |  | 27.055 |  | 20.702 |  |  |  |  |
|  | 26.522 |  | 17.951 |  |  |  |  |  |  | 27.222 |  | 20.735 |  |  |  |  |
| 2C | 26.91 | 26.883 | 18.826 | 18.845 | 8.038 |  | -1.259 | 2.39329793 | 2L | 30.603 | 30.6033333 | 22.159 | 22.2153333 | 8.388 | 1.956 | 0.25774208 |
|  | 30.41 |  | 18.864 |  |  |  |  |  |  | 30.519 |  | 22.218 |  |  |  |  |
|  | 26.856 |  | 19.617 |  |  |  |  |  |  | 30.688 |  | 22.269 |  |  |  |  |
| 3C | 27.2504253 | 27.137118 | 18.7992306 | 18.7992306 | 8.338 |  | -0.994 | 1.99169951 | 3L | 28.317 | 28.339 | 20.333 | 20.361 | 7.978 | 1.546 | 0.34245824 |
|  | 26.8293247 |  |  |  |  |  |  |  |  | 28.432 |  | 20.389 |  |  |  |  |
|  | 27.331604 |  |  |  |  |  |  |  |  | 28.268 |  | 20.609 |  |  |  |  |
| 4C | 29.3279438 | 29.2418702 | 23.6165199 | 20.2099085 | 9.032 |  | -0.25 | 1.18920712 | 4L | 26.682 | 26.6683333 | 18.004 | 18.065 | 8.603 | 2.171 | 0.2220567 |
|  | 29.1545353 |  | 20.2395496 |  |  |  |  |  |  | 26.677 |  | 17.955 |  |  |  |  |
|  | 29.2431316 |  | 20.1802673 |  |  |  |  |  |  | 26.646 |  | 18.236 |  |  |  |  |
| 5C | 29.1225834 | 29.0934493 | 19.4764881 | 19.4514109 | 9.642 |  | 0.36 | 0.77916458 | 5L | 26.424 | 26.3583333 | 17.386 | 17.35 | 9.008 | 2.576 | 0.16770528 |
|  | 29.007782 |  | 19.4643574 |  |  |  |  |  |  | 26.277 |  | 17.208 |  |  |  |  |
|  | 29.1499825 |  | 19.4133873 |  |  |  |  |  |  | 26.374 |  | 17.456 |  |  |  |  |
| 6C | 26.825201 | 26.8220272 | 19.0875683 | 18.8734118 | 7.949 |  | -1.333 | 2.51925996 | 6L | 24.909 | 24.7896667 | 16.465 | 16.6886667 | 8.101 | 1.669 | 0.31447124 |
|  | 26.8051929 |  | 18.6032295 |  |  |  |  |  |  | 24.686 |  | 16.812 |  |  |  |  |
|  | 26.8356876 |  | 18.9294376 |  |  |  |  |  |  | 24.774 |  | 16.789 |  |  |  |  |
| 7C | 28.1671753 | 28.1174634 | 20.1270046 | 20.1901512 | 7.927 |  | -1.355 | 2.55797116 | 7L | 27.851 | 27.817 | 18.553 | 18.355 | 9.462 | 3.03 | 0.12242754 |
|  | 28.0827789 |  | 20.2347374 |  |  |  |  |  |  | 27.715 |  | 18.456 |  |  |  |  |
|  | 28.1024361 |  | 20.2087116 |  |  |  |  |  |  | 27.885 |  | 18.056 |  |  |  |  |
| 8C | 26.845686 | 26.9710617 | 18.3640633 | 18.2693113 | 8.702 |  | -0.58 | 1.49484925 | 8L | 26.143 | 26.2703333 | 18.442 | 18.5513333 | 7.719 | 1.296 | 0.40725378 |
|  | 26.7785683 |  | 18.1762218 |  |  |  |  |  |  | 26.309 |  | 18.547 |  |  |  |  |
|  | 27.2889309 |  | 18.2676487 |  |  |  |  |  |  | 26.359 |  | 18.665 |  |  |  |  |
| 9C | 28.6336765 | 28.6249968 | 19.2814178 | 18.6610231 | 9.963 |  | 0.681 | 0.62373279 | 9L | 27.724 | 27.694 | 19.602 | 19.7266667 | 7.968 | 1.536 | 0.34484023 |
|  | 28.8786831 |  | 18.6596012 |  |  |  |  |  |  | 27.611 |  | 19.772 |  |  |  |  |
|  | 28.3626308 |  | 18.6624451 |  |  |  |  |  |  | 27.747 |  | 19.806 |  |  |  |  |
| 10C | 28.6123314 | 28.745376 | 16.9532909 | 17.0168451 | 11.728 |  | 2.446 | 0.18351883 | 10L | 25.463 | 25.463 | 18.073 | 18.164 | 7.299 | 0.867 | 0.54828579 |
|  | 28.9048901 |  | 16.9918766 |  |  |  |  |  |  | 25.383 |  | 18.066 |  |  |  |  |
|  | 28.7189064 |  | 17.1053677 |  |  |  |  |  |  | 25.496 |  | 18.353 |  |  |  |  |
| 11c | 28.5820904 | 28.5092449 | 18.8971291 | 18.8971 | 9.612 |  | 0.33 | 0.79553648 | 11L | 25.697 | 25.638 | 17.682 | 17.8163333 | 7.822 | 1.39 | 0.3815648 |
|  | 28.5875187 |  |  |  |  |  |  |  |  | 25.591 |  | 17.842 |  |  |  |  |
|  | 28.3581257 |  |  |  |  |  |  |  |  | 25.626 |  | 17.925 |  |  |  |  |
| 12C | 27.2385712 | 26.9914799 | 17.2714615 | 17.2213046 | 9.77 |  | 0.488 | 0.71301286 | 12L | 25.453 | 25.538 | 18.346 | 18.3106667 | 7.228 | 0.856 | 0.55248224 |
|  | 26.8014488 |  | 17.1660061 |  |  |  |  |  |  | 25.515 |  | 18.206 |  |  |  |  |
|  | 26.9344196 |  | 17.2264462 |  |  |  |  |  |  | 25.646 |  | 18.38 |  |  |  |  |
| 13C | 26.6439686 | 26.49296 | 17.7740536 | 17.849268 | 8.643 |  | -0.639 | 1.55724938 | 13L | 28.106 | 28.136 | 19.63 | 19.682 | 8.454 | 2.022 | 0.24621661 |
|  | 26.4465141 |  | 17.8227177 |  |  |  |  |  |  | 27.935 |  | 19.699 |  |  |  |  |
|  | 26.3883972 |  | 17.9510326 |  |  |  |  |  |  | 28.367 |  | 19.717 |  |  |  |  |
| 14C | 28.0150948 | 28.0279675 | 17.538435 | 17.5721436 | 10.457 |  | 1.175 | 0.44288376 | 14L | 26.936 | 27.0963333 | 18.172 | 18.3716667 | 8.725 | 2.293 | 0.20405076 |
|  | 28.119585 |  | 17.4516945 |  |  |  |  |  |  | 26.946 |  | 18.376 |  |  |  |  |
|  | 27.9492226 |  | 17.7263012 |  |  |  |  |  |  | 27.407 |  | 18.567 |  |  |  |  |
| 15C | 25.5075989 | 25.7040965 | 18.0387783 | 18.0091597 | 7.695 |  | -1.587 | 3.00423985 | 15L | 26.17 | 26.2626667 | 18.499 | 18.508 | 7.754 | 1.322 | 0.39998006 |
|  | 25.6811199 |  | 17.920578 |  |  |  |  |  |  | 26.19 |  | 18.351 |  |  |  |  |
|  | 25.9235706 |  | 18.0681229 |  |  |  |  |  |  | 26.428 |  | 18.674 |  |  |  |  |
| 16C | 27.878212 | 27.854538 | 17.3989792 | 17.4165122 | 10.438 |  | 1.156 | 0.44875503 | 16L | 29.264 | 29.2313333 | 18.821 | 18.9006667 | 10.331 | 3.899 | 0.06703229 |
|  | 27.773735 |  | 17.3921356 |  |  |  |  |  |  | 29.072 |  | 19.044 |  |  |  |  |
|  | 27.9116669 |  | 17.4584217 |  |  |  |  |  |  | 29.358 |  | 18.837 |  |  |  |  |
| 17C | 27.8546543 | 27.8569825 | 18.6168537 | 18.6856136 | 9.171 |  | -0.111 | 1.07997656 | 17L | 27.865 | 27.8256667 | 18.36 | 18.2436667 | 9.582 | 3.15 | 0.11265631 |
|  | 27.8346596 |  | 18.7170601 |  |  |  |  |  |  | 27.792 |  | 18.137 |  |  |  |  |
|  | 27.8816338 |  | 18.7229271 |  |  |  |  |  |  | 27.82 |  | 18.234 |  |  |  |  |
| 18C | 28.6770039 | 28.4658833 | 17.9625759 | 17.9248327 | 10.541 |  | 1.259 | 0.41783348 | 18L | 28.087 | 27.4146667 | 18.091 | 17.9536667 | 9.461 | 3.029 | 0.12251243 |
|  | 28.4477787 |  | 17.9196835 |  |  |  |  |  |  | 27.938 |  | 17.888 |  |  |  |  |
|  | 28.2728672 |  | 17.8922386 |  |  |  |  |  |  | 26.219 |  | 17.882 |  |  |  |  |
| 19C | 27.654 | 27.6723333 | 17.712 | 17.7906667 | 9.882 |  | 0.6 | 0.65975396 | 19L | 26.743 | 26.6683333 | 18.206 | 18.854 | 7.814 | 1.382 | 0.38368652 |
|  | 27.758 |  | 17.779 |  |  |  |  |  |  | 26.537 |  | 19.17 |  |  |  |  |
|  | 27.605 |  | 17.881 |  |  |  |  |  |  | 26.725 |  | 18.538 |  |  |  |  |
| 20C | 29.657 | 29.6273333 | 18.057 | 18.0833333 | 11.544 |  | 2.262 | 0.20848276 | 20L | 26.761 | 26.725 | 18.906 | 19.2043333 | 7.521 | 1.089 | 0.4700871 |
|  | 29.587 |  | 18.126 |  |  |  |  |  |  | 26.707 |  | 19.213 |  |  |  |  |
|  | 29.638 |  | 18.067 |  |  |  |  |  |  | 26.707 |  | 19.494 |  |  |  |  |
| 21C | 28.118 | 28.0835 | 17.072 | 17.0153333 | 11.068 |  | 1.786 | 0.28997491 | 21L | 28.372 | 28.2466667 | 18.181 | 18.2786667 | 9.968 | 3.536 | 0.08621006 |
|  | 28.049 |  | 17.022 |  |  |  |  |  |  | 28.123 |  | 18.457 |  |  |  |  |
|  | 29.081 |  | 16.952 |  |  |  |  |  |  | 28.245 |  | 18.198 |  |  |  |  |
| 22C | 28.471 | 28.485 | 17.536 | 17.359 | 11.126 |  | 1.844 | 0.27854841 | 22L | 27.226 | 27.214 | 17.569 | 17.6836667 | 9.531 | 3.099 | 0.11670999 |
|  | 28.425 |  | 17.28 |  |  |  |  |  |  | 27.291 |  | 17.765 |  |  |  |  |
|  | 28.559 |  | 17.261 |  |  |  |  |  |  | 27.125 |  | 17.717 |  |  |  |  |
| 23C | 29.674 | 29.501 | 19.602 | 19.6466667 | 9.855 |  | 0.573 | 0.6722175 | 23L | 31.102 |  | 19.131 | 19.0896667 | 9.429 | 2.997 | 0.1252602 |
|  | 29.477 |  | 19.748 |  |  |  |  |  |  | 28.452 | 28.518 | 19.104 |  |  |  |  |
|  | 29.352 |  | 19.59 |  |  |  |  |  |  | 28.584 |  | 19.034 |  |  |  |  |
| 24C | 30.332 | 30.494 | 18.372 | 18.4146667 | 12.08 |  | 2.798 | 0.14378649 | 24L | 30.502 | 30.475 | 21.572 | 21.661 | 8.814 | 2.382 | 0.19184326 |
|  | 30.527 |  | 18.35 |  |  |  |  |  |  | 27.894 |  | 21.883 |  |  |  |  |
|  | 30.623 |  | 18.522 |  |  |  |  |  |  | 30.445 |  | 21.528 |  |  |  |  |
| 25C | 29.452 | 29.4046667 | 17.725 | 17.789 | 11.615 |  | 2.333 | 0.19847098 | 25L | 28.36 | 28.151 | 18.503 | 18.539 | 9.612 | 3.18 | 0.11033787 |
|  | 29.404 |  | 17.764 |  |  |  |  |  |  | 26.54 |  | 18.446 |  |  |  |  |
|  | 29.358 |  | 17.878 |  |  |  |  |  |  | 27.942 |  | 18.668 |  |  |  |  |
| 26C | 29.187 | 29.183 | 18.711 | 18.182 | 11.001 |  | 1.719 | 0.3037592 | 26L | 27.489 | 27.4493333 | 18.133 | 18.2383333 | 9.211 | 2.779 | 0.14569265 |
|  | 28.882 |  | 18.107 |  |  |  |  |  |  | 27.758 |  | 18.2 |  |  |  |  |
|  | 29.48 |  | 18.257 |  |  |  |  |  |  | 27.101 |  | 18.382 |  |  |  |  |
| 27C | 29.63 | 29.6373333 | 17.708 | 17.7473333 | 11.89 |  | 2.608 | 0.16402641 | 27L | 27.481 | 27.312 | 17.887 | 18.516 | 8.796 | 2.364 | 0.19425182 |
|  | 29.644 |  | 17.627 |  |  |  |  |  |  | 25.547 |  | 18.29 |  |  |  |  |
|  | 29.638 |  | 17.907 |  |  |  |  |  |  | 27.143 |  | 18.742 |  |  |  |  |
| 28C | 27.788 | 27.7123333 | 16.846 | 16.9256667 | 10.787 |  | 1.505 | 0.35233019 | 28L | 25.545 | 25.502 | 16.92 | 16.6813333 | 8.821 | 2.389 | 0.19091469 |
|  | 27.767 |  | 16.919 |  |  |  |  |  |  | 27.327 |  | 16.215 |  |  |  |  |
|  | 27.582 |  | 17.012 |  |  |  |  |  |  | 25.46 |  | 16.909 |  |  |  |  |
| 29C | 27.952 | 28.0443333 | 17.277 | 17.201 | 10.843 |  | 1.561 | 0.33891608 | 29L | 28.048 | 27.7166667 | 17.536 | 17.7283333 | 9.988 | 3.556 | 0.08502318 |
|  | 28.068 |  | 17.164 |  |  |  |  |  |  | 27.295 |  | 17.837 |  |  |  |  |
|  | 28.113 |  | 17.162 |  |  |  |  |  |  | 27.807 |  | 17.812 |  |  |  |  |
