# Supplementary material for: The Expression and Prognostic Value of FGF2, FGFR3, and FGFBP1 in Esophageal Squamous Cell Carcinoma
Source: Anal Cell Pathol (Amst). 2020 Dec 11;2020:2872479. doi: 10.1155/2020/2872479 (PMC7748917; doi:10.1155/2020/2872479)
Supplement: Supplementary materials — Table 1(s)-Table 3(s): the data of immunohistochemical patients. Table 4 s-7 s: mPCR data information. [file 2872479.f1.zip › Table 6s.docx]

| Table 6s.FGFBP1-mRNA | | | | | | | | | | | | | | | |
| --- | --- | --- | --- | --- | --- | --- | --- | --- | --- | --- | --- | --- | --- | --- | --- |
| sample | primer-FGFBP1 | cancer-CT mean | actin | CT mean | CT FGFBP1-CTactin | ΔΔCT | 2-ΔΔCT | sample | primer-FGFBP1 | normal-CT mean | actin | CT mean | CT FGFBP1-CTactin | ΔΔCT | 2-ΔΔCT |
| 1C | 37.525 | 37.525 |  | 22.51 | 15.015 | 0 | 1 | 1L | 34.515 | 35.5185 |  | 22.483 | 13.0355 | 0 | 1 |
|  | 35.94 |  |  |  |  |  |  |  | 35.202 |  |  |  |  |  |  |
|  |  |  |  |  |  |  |  |  | 35.835 |  |  |  |  |  |  |
| 2C | 33.953 | 33.9545 |  | 20.812 | 13.1425 | -1.8725 | 3.66166549 | 2L | 35.596 | 34.554 |  | 19.901 | 14.653 | 1.6175 | 0.32589972 |
|  | 33.956 |  |  |  |  |  |  |  | 33.782 |  |  |  |  |  |  |
|  |  |  |  |  |  |  |  |  | 34.284 |  |  |  |  |  |  |
| 3C | 33.828 | 33.828 |  | 20.162 | 13.666 | -1.349 | 2.54735495 | 3L | 37.967 | 36.704 |  | 21.129 | 15.575 | 2.5395 | 0.17200233 |
|  | 34.07 |  |  |  |  |  |  |  | 35.441 |  |  |  |  |  |  |
|  | 34.336 |  |  |  |  |  |  |  |  |  |  |  |  |  |  |
| 4C | 34.974 | 34.8255 |  | 18.177 | 16.6485 | 1.6335 | 0.32230534 | 4L | 33.899 | 33.475 |  | 18.536 | 14.939 | 1.9035 | 0.26729412 |
|  | 34.677 |  |  |  |  |  |  |  | 33.314 |  |  |  |  |  |  |
|  |  |  |  |  |  |  |  |  | 33.212 |  |  |  |  |  |  |
| 5C | 33.957 | 32.973 |  | 17.914 | 15.059 | 0.044 | 0.96996191 | 5L | 35.129 | 34.0943333 |  | 17.874 | 16.2203333 | 3.18483333 | 0.10996884 |
|  | 32.974 |  |  |  |  |  |  |  | 33.028 |  |  |  |  |  |  |
|  | 32.972 |  |  |  |  |  |  |  | 34.126 |  |  |  |  |  |  |
| 6C | 31.015 | 31.4103333 |  | 18.399 | 13.0113333 | -2.0036667 | 4.01017909 | 6L | 34.531 | 34.5196667 |  | 17.816 | 16.7036667 | 3.66816667 | 0.07866324 |
|  | 31.552 |  |  |  |  |  |  |  | 35.214 |  |  |  |  |  |  |
|  | 31.664 |  |  |  |  |  |  |  | 33.814 |  |  |  |  |  |  |
| 7C | 33.885 | 33.651 |  | 19.61 | 14.041 | -0.974 | 1.96427919 | 7L | 34.242 | 34.3006667 |  | 18.209 | 16.0916667 | 3.05616667 | 0.12022704 |
|  | 33.309 |  |  |  |  |  |  |  | 34.283 |  |  |  |  |  |  |
|  | 33.759 |  |  |  |  |  |  |  | 34.377 |  |  |  |  |  |  |
| 8C | 32.137 | 32.316 |  | 15.555 | 16.761 | 1.746 | 0.29812722 | 8L | 33.021 | 32.9536667 |  | 18.206 | 14.7476667 | 1.71216667 | 0.30520137 |
|  | 32.495 |  |  |  |  |  |  |  | 32.955 |  |  |  |  |  |  |
|  |  |  |  |  |  |  |  |  | 32.885 |  |  |  |  |  |  |
| 9C | 32.671 | 33.3985 |  | 18.583 | 14.8155 | -0.1995 | 1.14830032 | 9L | 32.553 | 32.724 |  | 18.52 | 14.204 | 1.1685 | 0.44488365 |
|  | 33.397 |  |  |  |  |  |  |  | 32.89 |  |  |  |  |  |  |
|  | 33.4 |  |  |  |  |  |  |  | 32.729 |  |  |  |  |  |  |
| 10C | 31.777 | 31.354 |  | 14.996 | 16.358 | 1.343 | 0.39420009 | 10L | 36.574 | 36.05 |  | 18.615 | 17.435 | 4.3995 | 0.04738256 |
|  | 30.595 |  |  |  |  |  |  |  | 35.981 |  |  |  |  |  |  |
|  | 31.69 |  |  |  |  |  |  |  | 35.595 |  |  |  |  |  |  |
| 11c | 35.096 | 35.0595 |  | 19.697 | 15.3625 | 0.3475 | 0.78594486 | 11L | 34.847 | 35.14 |  | 19.198 | 15.942 | 2.9065 | 0.13336944 |
|  | 35.023 |  |  |  |  |  |  |  | 35.619 |  |  |  |  |  |  |
|  |  |  |  |  |  |  |  |  | 34.954 |  |  |  |  |  |  |
| 12C | 31.543 | 31.3696667 |  | 20.929 | 10.4406667 | -4.5743333 | 23.8238279 | 12L | 32.981 | 31.82 |  | 18.314 | 13.506 | 0.4705 | 0.72171443 |
|  | 31.238 |  |  |  |  |  |  |  | 31.716 |  |  |  |  |  |  |
|  | 31.328 |  |  |  |  |  |  |  | 31.924 |  |  |  |  |  |  |
| 13C | 34.97 | 34.47 |  | 17.942 | 16.528 | 1.513 | 0.35038186 | 13L | 35.34 | 34.961 |  | 19.425 | 15.536 | 2.5005 | 0.17671544 |
|  | 33.97 |  |  |  |  |  |  |  | 34.658 |  |  |  |  |  |  |
|  |  |  |  |  |  |  |  |  | 34.885 |  |  |  |  |  |  |
| 14C | 34.32 | 34.503 |  | 17.816 | 16.687 | 1.672 | 0.313818 | 14L | 35.714 | 35.4835 |  | 18.103 | 17.3805 | 4.345 | 0.04920675 |
|  | 34.686 |  |  |  |  |  |  |  | 35.253 |  |  |  |  |  |  |
|  |  |  |  |  |  |  |  |  | 33.397 |  |  |  |  |  |  |
| 15C | 34.501 | 34.37 |  | 18.053 | 16.317 | 1.302 | 0.40556358 | 15L | 34.263 | 34.2213333 |  | 17.27 | 16.9513333 | 3.91583333 | 0.0662547 |
|  | 34.239 |  |  |  |  |  |  |  | 34.212 |  |  |  |  |  |  |
|  |  |  |  |  |  |  |  |  | 34.189 |  |  |  |  |  |  |
| 16C | 34.021 | 34.08 |  | 17.914 | 16.166 | 1.151 | 0.45031299 | 16L | 38.049 | 37.9025 |  | 19.21 | 18.6925 | 5.657 | 0.01981862 |
|  | 34.139 |  |  |  |  |  |  |  | 37.756 |  |  |  |  |  |  |
|  |  |  |  |  |  |  |  |  |  |  |  |  |  |  |  |
| 17C | 30.75 | 30.651 |  | 17.056 | 13.595 | -1.42 | 2.67585511 | 17L | 34.182 | 34.385 |  | 19.517 | 14.868 | 1.8325 | 0.28077765 |
|  | 30.78 |  |  |  |  |  |  |  | 34.588 |  |  |  |  |  |  |
|  | 30.423 |  |  |  |  |  |  |  |  |  |  |  |  |  |  |
| 18C | 34.991 | 34.621 |  | 20.759 | 13.862 | -1.153 | 2.22375832 | 18L | 32.632 | 32.7346667 |  | 18.83 | 13.9046667 | 0.86916667 | 0.54746299 |
|  | 34.374 |  |  |  |  |  |  |  | 32.952 |  |  |  |  |  |  |
|  | 34.498 |  |  |  |  |  |  |  | 32.62 |  |  |  |  |  |  |
| 19C | 32.885 | 33.0916667 |  | 19.718 | 13.3736667 | -1.6413333 | 3.11954005 | 19L | 35.221 | 35.4243333 |  | 19.881 | 15.5433333 | 2.50783333 | 0.17581946 |
|  | 33.218 |  |  |  |  |  |  |  | 35.486 |  |  |  |  |  |  |
|  | 33.172 |  |  |  |  |  |  |  | 35.566 |  |  |  |  |  |  |
| 20C | 38.453 | 37.002 |  | 18.433 | 18.569 | 3.554 | 0.08514113 | 20L | 33.542 | 33.4705 |  | 19.397 | 14.0735 | 1.038 | 0.48700213 |
|  | 36.703 |  |  |  |  |  |  |  | 33.399 |  |  |  |  |  |  |
|  | 37.301 |  |  |  |  |  |  |  |  |  |  |  |  |  |  |
| 21C | 34.325 | 34.5203333 |  | 16.942 | 17.5783333 | 2.56333333 | 0.16918419 | 21L | 33.542 | 33.4705 |  | 19.397 | 14.0735 | 1.038 | 0.48700213 |
|  | 34.722 |  |  |  |  |  |  |  | 33.399 |  |  |  |  |  |  |
|  | 34.514 |  |  |  |  |  |  |  |  |  |  |  |  |  |  |
| 22C | 34.723 | 33.032 |  | 17.075 | 15.957 | 0.942 | 0.5205108 | 22L | 32.811 | 32.765 |  | 19.069 | 13.696 | 0.6605 | 0.632659 |
|  | 33.449 |  |  |  |  |  |  |  | 32.719 |  |  |  |  |  |  |
|  | 32.615 |  |  |  |  |  |  |  |  |  |  |  |  |  |  |
| 23C | 36.292 | 36.8575 |  | 25.491 | 11.3665 | -3.6485 | 12.5403004 | 23L | 31.72 | 32.0195 |  | 20.418 | 11.6015 | -1.434 | 2.70194817 |
|  | 37.423 |  |  |  |  |  |  |  | 32.319 |  |  |  |  |  |  |
|  |  |  |  |  |  |  |  |  |  |  |  |  |  |  |  |
| 24C | 30.615 | 30.3956667 |  | 17.914 | 12.4816667 | -2.5333333 | 5.78907695 | 24L | 31.251 | 31.292 |  | 23.029 | 8.263 | -4.7725 | 27.3316376 |
|  | 30.254 |  |  |  |  |  |  |  | 31.333 |  |  |  |  |  |  |
|  | 30.318 |  |  |  |  |  |  |  |  |  |  |  |  |  |  |
| 25C | 32.979 | 32.7433333 |  | 16.661 | 16.0823333 | 1.06733333 | 0.47720024 | 25L | 31.705 | 31.7396667 |  | 20.073 | 11.6666667 | -1.3688333 | 2.58261633 |
|  | 32.438 |  |  |  |  |  |  |  | 31.567 |  |  |  |  |  |  |
|  | 32.813 |  |  |  |  |  |  |  | 31.947 |  |  |  |  |  |  |
| 26C | 33.11 | 32.8343333 |  | 18.011 | 14.8233333 | -0.1916667 | 1.14208234 | 26L | 32.07 | 32.02 |  | 19.615 | 12.405 | -0.6305 | 1.54810143 |
|  | 32.977 |  |  |  |  |  |  |  | 31.97 |  |  |  |  |  |  |
|  | 32.416 |  |  |  |  |  |  |  |  |  |  |  |  |  |  |
| 27C | 32.508 | 32.6155 |  | 17.922 | 14.6935 | -0.3215 | 1.24962914 | 27L | 33.941 | 33.9415 |  | 19.251 | 14.6905 | 1.655 | 0.31753775 |
|  | 32.723 |  |  |  |  |  |  |  | 33.942 |  |  |  |  |  |  |
|  |  |  |  |  |  |  |  |  |  |  |  |  |  |  |  |
| 28C | 31.339 | 31.302 |  | 15.424 | 15.878 | 0.863 | 0.54980807 | 28L | 35.037 | 35.314 |  | 19.237 | 16.077 | 3.0415 | 0.12145552 |
|  | 30.654 |  |  |  |  |  |  |  | 35.591 |  |  |  |  |  |  |
|  | 31.913 |  |  |  |  |  |  |  |  |  |  |  |  |  |  |
| 29C | 33.656 | 32.899 |  | 17.464 | 15.435 | 0.42 | 0.74742462 | 29L | 33.471 | 33.6746667 |  | 18.415 | 15.2596667 | 2.22416667 | 0.21402234 |
|  | 32.142 |  |  |  |  |  |  |  | 33.756 |  |  |  |  |  |  |
|  |  |  |  |  |  |  |  |  | 33.797 |  |  |  |  |  |  |
