# Supplementary material for: The Expression and Prognostic Value of FGF2, FGFR3, and FGFBP1 in Esophageal Squamous Cell Carcinoma
Source: Anal Cell Pathol (Amst). 2020 Dec 11;2020:2872479. doi: 10.1155/2020/2872479 (PMC7748917; doi:10.1155/2020/2872479)
Supplement: Supplementary materials — Table 1(s)-Table 3(s): the data of immunohistochemical patients. Table 4 s-7 s: mPCR data information. [file 2872479.f1.zip › Table 7s primer.docx]

| Gene ID | The name of the primer | Primer sequences（5' -3'） | The length  bp | Tm | GC% | Product  bp |
| --- | --- | --- | --- | --- | --- | --- |
| 2247 | FGF2-F1 | TTCAAGCAGAAGAGAGAGGAG | 21 | 56.76 | 47.62 | 106 |
|  | FGF2-R1 | TCCGTAACACATTTAGAAGCC | 21 | 55.99 | 42.86 |  |
| 2261 | FGFR3-F1 | ACCAAGCCTGTCACCGTAG | 19 | 59.33 | 57.89 | 204 |
|  | FGFR3-R1 | CAGAAACTCCCGCAGGTTACC | 21 | 60.94 | 57.14 |  |
| 9982 | FGFBP1-F1 | GGGAGGAGCTGTGAGTAACG | 20 | 59.83 | 60.00 | 140 |
|  | FGFBP1-R1 | CAGGCAGTGCGAGTGAATTG | 20 | 59.83 | 55.00 |  |
