# Supplementary material for: The Expression and Prognostic Value of FGF2, FGFR3, and FGFBP1 in Esophageal Squamous Cell Carcinoma
Source: Anal Cell Pathol (Amst). 2020 Dec 11;2020:2872479. doi: 10.1155/2020/2872479 (PMC7748917; doi:10.1155/2020/2872479)
Supplement: Supplementary materials — Table 1(s)-Table 3(s): the data of immunohistochemical patients. Table 4 s-7 s: mPCR data information. [file 2872479.f1.zip › Table2s.docx]

| Table 2s Immunohistochemical statistics | | | | | | | | | | | | | | | | | | | | | | |
| --- | --- | --- | --- | --- | --- | --- | --- | --- | --- | --- | --- | --- | --- | --- | --- | --- | --- | --- | --- | --- | --- | --- |
| Name | Race | Age (years) | Sex | Tumor site | Tumor size | specimen type | Differentiation | Infiltrating depth | TNM（The eighth edition） | AJCC | Lymph metastasis | Lymph metastasis | Vascular invasion | Vascular invasion | 神经侵犯 | 神经侵犯 | 0S(最新） | PFS | 生存状态 | FGF2 | FGFR3 | FGFBP1 |
| 努哈·梯也拜 | hazak | 70 | male | Middle | 4 | Medullary type | well | Muscular layer | ⅡA | 1 | negative | 0 | negative | 0 | negative | 0 | 40 | 7 | 1 | + | + | + |
| 唐明基 | han | 68 | male | Middle | 2.2 | Ulcer type | Poor | submucous | ⅡB | 2 | positive | 0 | negative | 0 | negative | 0 | 36 | 31 | 1 | + | + | + |
| 努尔哈山 | hazak | 67 | male | Lower | 3.8 | Ulcer type | Poor | The outer membrane | IIIC | 1 | negative | 1 | negative | 0 | negative | 0 | 36 | 36 | 1 | + | - | - |
| 那万·扎勒扎汗 | hazak | 61 | male | Lower | 5 | Ulcer type | well | Muscular layer | ⅠB | 0 | negative | 0 | negative | 0 | negative | 0 | 64 | 12 | 0 | + | + | + |
| 沈延龙 | han | 71 | male | Lower | 5 | Mushroom umbrella | Moderate | Muscular layer | ⅢA | 2 | positive | 1 | positive | 1 | negative | 0 | 3 | 3 | 1 | + | + | + |
| 阿比江·马木里别克 | hazak | 70 | female | Lower | 5 | Ulcer type | Moderate | The outer membrane | ⅡA | 1 | negative | 0 | negative | 0 | negative | 0 | 12 | 12 | 1 | - | - | - |
| 王兴善 | han | 70 | male | Middle | 6 | Ulcer type | well | The outer membrane | ⅡA | 1 | negative | 0 | positive | 1 | positive | 1 | 2 | 2 | 1 | + | + | + |
| 佐尔古丽·叶明纳洪 | hazak | 53 | female | Middle | 2 | Ulcer type | Moderate | The outer membrane | ⅡB | 1 | negative | 0 | negative | 0 | negative | 0 | 63 | 10 | 0 | + | + | + |
| 付思喜 | han | 38 | male | Lower | 4 | Ulcer type | Poor | The outer membrane | ⅡA | 1 | negative | 0 | negative | 0 | negative | 0 | 50 | 41 | 1 | - | - | - |
| 邹克宽 | han | 58 | male | Lower | 4.3 | Ulcer type | Poor | The outer membrane | IIIC | 2 | positive | 1 | positive | 1 | negative | 0 | 30 | 4 | 1 | + | + | + |
| 那孜尔汗·胡那甫亚 | hazak | 67 | male | Middle | 2 | Ulcer type | Moderate | submucous | ⅠB | 0 | negative | 0 | negative | 0 | negative | 0 | 36 | 34 | 1 | - | - | - |
| 努尔肯 | hazak | 43 | male | Middle | 3.8 | Ulcer type | Moderate | Muscular layer | ⅡB | 1 | negative | 0 | negative | 0 | positive | 1 | 38 | 1 | 1 | + | + | + |
| 倪修玉 | han | 69 | male | Middle | 4.5 | Ulcer type | Poor | The outer membrane | ⅡB | 1 | negative | 0 | negative | 0 | positive | 1 | 12 | 7 | 1 | + | + | + |
| 王爱华 | han | 70 | female | Lower | 4.7 | Mushroom umbrella | Poor | The outer membrane | ⅢB | 2 | positive | 1 | negative | 0 | negative | 0 | 36 | 12 | 1 | + | + | + |
| 曹云英 | han | 72 | female | Lower | 4 | Medullary type | Moderate | Muscular layer | ⅡB | 1 | positive | 1 | negative | 0 | negative | 0 | 12 | 12 | 1 | + | + | + |
| 黄长平 | han | 55 | male | Lower | 6 | Ulcer type | Poor | Muscular layer | ⅢA | 2 | positive | 1 | negative | 0 | negative | 0 | 8 | 8 | 1 | + | + | + |
| 丁邦明 | han | 78 | male | Middle | 3.5 | Mushroom umbrella | Poor | Muscular layer | ⅡB | 1 | negative | 0 | positive | 1 | negative | 0 | 12 | 12 | 1 | + | + | + |
| 赵兴菊 | han | 43 | female | Middle | 5.5 | Medullary type | Moderate | The outer membrane | ⅡB | 1 | negative | 0 | negative | 0 | negative | 0 | 48 | 45 | 1 | - | - | - |
| 梁秀英 | han | 64 | female | Upper | 2.5 | Ulcer type | Poor | submucous | ⅠB | 0 | negative | 0 | negative | 0 | negative | 0 | 48 | 48 | 1 | - | - | - |
| 肯杰依·萨尔恰 | hazak | 72 | female | Middle | 5.5 | Ulcer type | well | Muscular layer | ⅡA | 1 | negative | 0 | negative | 0 | negative | 0 | 1 | 1 | 1 | + | + | + |
| 张军宣 | han | 60 | male | Lower | 2.6 | Uplift type | Moderate | The outer membrane | ⅡA | 1 | negative | 0 | negative | 0 | negative | 0 | 48 | 20 | 1 | - | + | - |
| 那比汗·阿散 | hazak | 57 | male | Lower | 5 | Ulcer type | Poor | The outer membrane | ⅡB | 1 | negative | 0 | negative | 0 | negative | 0 | 58 | 35 | 0 | - | - | - |
| 郭树芳 | han | 60 | male | Lower | 4 | Ulcer type | Moderate | Muscular layer | ⅢA | 2 | positive | 1 | negative | 0 | negative | 0 | 36 | 26 | 1 | + | + | + |
| 吾拉孜别克·胡安尼西 | hazak | 79 | male | Lower | 4.5 | Ulcer type | well | Muscular layer | ⅢA | 2 | positive | 1 | negative | 0 | positive | 1 | 10 | 1 | 1 | + | + | + |
| 阿依达克·巴依丹 | hazak | 54 | female | Middle | 3 | Uplift type | Moderate | The outer membrane | ⅡB | 1 | negative | 0 | negative | 0 | negative | 0 | 1 | 12 | 1 | + | + | + |
| 王菊梅 | han | 62 | female | Middle | 5 | Ulcer type | Moderate | Muscular layer | ⅡB | 1 | positive | 1 | negative | 0 | negative | 0 | 16 | 14 | 1 | + | + | + |
| 梁成伟 | han | 83 | male | Lower | 2 | Ulcer type | Poor | Muscular layer | ⅡA | 1 | negative | 0 | positive | 1 | negative | 0 | 2 | 2 | 1 | + | + | + |
| 金作星 | han | 81 | male | Middle | 3 | Medullary type | Poor | The outer membrane | ⅡB | 1 | negative | 0 | negative | 0 | negative | 0 | 18 | 12 | 1 | + | + | + |
| 古丽太·合孜尔别克 | hazak | 59 | female | Middle | 4 | Ulcer type | Moderate | The outer membrane | IIB | 2 | positive | 1 | positive | 1 | negative | 0 | 40 | 3 | 1 | - | - | - |
| 金恩斯·卡力木 | hazak | 56 | male | Lower | 7 | Ulcer type | Poor | The outer membrane | IIA | 2 | negative | 0 | negative | 0 | positive | 1 | 36 | 34 | 1 | - | - | - |
| 张全山 | han | 71 | male | Middle | 4.2 | Uplift type | Moderate | Muscular layer | ⅡB | 1 | negative | 0 | negative | 0 | negative | 0 | 1 | 1 | 1 | + | + | + |
| 李林书 | han | 47 | male | Lower | 5.5 | Ulcer type | Poor | The outer membrane | ⅡA | 1 | negative | 0 | negative | 0 | positive | 1 | 47 | 38 | 1 | - | - | - |
| 候国山 | han | 74 | male | Lower | 4.5 | Medullary type | Moderate | Muscular layer | ⅡA | 1 | negative | 0 | negative | 0 | negative | 0 | 55 | 55 | 0 | - | - | - |
| 赛依佬拜克·吐马尔拜克 | hazak | 60 | male | Middle | 2.3 | Medullary type | Moderate | Muscular layer | ⅡB | 1 | negative | 0 | negative | 0 | negative | 0 | 54 | 6 | 0 | + | + | - |
| 丁连丰 | han | 76 | male | Middle | 2.5 | Ulcer type | Moderate | Muscular layer | ⅡB | 1 | negative | 0 | negative | 0 | negative | 0 | 12 | 12 | 1 | + | + | + |
| 周明荣 | han | 57 | male | Lower | 3.5 | Ulcer type | Moderate | The outer membrane | ⅢB | 2 | positive | 1 | positive | 1 | negative | 0 | 20 | 20 | 1 | - | - | - |
| 常生浩 | han | 58 | male | Lower | 5.5 | Ulcer type | well | Muscular layer | ⅠB | 0 | negative | 0 | negative | 0 | negative | 0 | 10 | 10 | 1 | + | + | + |
| 铁列干·塔力甫拜 | hazak | 61 | male | Lower | 5 | Ulcer type | Moderate | The outer membrane | ⅡA | 1 | negative | 0 | negative | 0 | negative | 0 | 52 | 18 | 0 | + | - | - |
| 谭建明 | han | 64 | male | Lower | 4 | Uplift type | well | The outer membrane | ⅠB | 1 | negative | 0 | negative | 0 | negative | 0 | 40 | 36 | 1 | - | - | - |
| 乌拉别克·依拉合买提 | hazak | 61 | male | Middle | 3 | Ulcer type | Poor | The outer membrane | ⅢA | 2 | positive | 1 | negative | 0 | negative | 0 | 18 | 18 | 1 | - | - | - |
| 郑朝祥 | han | 73 | male | Middle | 4 | Ulcer type | Moderate | The outer membrane | ⅡB | 1 | negative | 0 | negative | 0 | negative | 0 | 2 | 2 | 1 | + | + | + |
| 唐仁凤 | han | 76 | male | Middle | 1.8 | Ulcer type | Moderate | The outer membrane | ⅡB | 1 | negative | 0 | negative | 0 | negative | 0 | 38 | 20 | 1 | - | - | - |
| 张贵芝 | han | 73 | female | Upper | 1 | Ulcer type | Poor | submucous | IB | 2 | positive | 1 | negative | 0 | negative | 0 | 36 | 2 | 1 | + | + | + |
| 萨勒木·阿依达尔汗 | hazak | 76 | male | Lower | 3.5 | Mushroom umbrella | well | Muscular layer | ⅠB | 0 | negative | 0 | negative | 0 | negative | 0 | 52 | 35 | 0 | + | + | + |
| 阿布扎力汗·马克赞 | hazak | 73 | male | Lower | 2 | Medullary type | Poor | The outer membrane | ⅡA | 1 | negative | 0 | negative | 0 | negative | 0 | 40 | 36 | 1 | - | + | - |
| 李定宛 | han | 54 | male | Lower | 1.5 | Ulcer type | Moderate | Muscular layer | ⅡA | 1 | negative | 0 | negative | 0 | negative | 0 | 40 | 25 | 1 | - | - | + |
| 哈斯木汗 | hazak | 64 | male | Upper段 | 3 | Ulcer type | well | 深Muscular layer | IIB | 1 | negative | 0 | negative | 0 | negative | 0 | 72 | 72 | 0 | - | - | - |
| 阿尼吾尔·萨里亚很 | hazak | 57 | male | Middle | 2.6 | Ulcer type | Moderate | The outer membrane | ⅡB | 1 | negative | 0 | negative | 0 | positive | 1 | 36 | 12 | 1 | + | + | + |
