# Supplementary material for: The Expression and Prognostic Value of FGF2, FGFR3, and FGFBP1 in Esophageal Squamous Cell Carcinoma
Source: Anal Cell Pathol (Amst). 2020 Dec 11;2020:2872479. doi: 10.1155/2020/2872479 (PMC7748917; doi:10.1155/2020/2872479)
Supplement: Supplementary materials — Table 1(s)-Table 3(s): the data of immunohistochemical patients. Table 4 s-7 s: mPCR data information. [file 2872479.f1.zip › Table5s.docx]

| Table5s .FGFR3-mRNA | | | | | | | | | | | | | | | |
| --- | --- | --- | --- | --- | --- | --- | --- | --- | --- | --- | --- | --- | --- | --- | --- |
| sample | primer-FGFR3 | Cancer-CT mean | actin | CT mean | CT FGFR3-CTactin | ΔΔCT | 2-ΔΔCT | sample | primer-FGFR3 | normal-CT mean | actin | CT mean | CT FGFR3-Ctactin | ΔΔCT | 2-ΔΔCT |
| 1C | 32.173 | 32.173 | 19.901 |  | 12.272 | 0 | 1 | 1L | 27.948 | 27.8555 |  | 22.523 | 5.3325 | 0 | 1 |
|  | 33.054 |  |  |  |  |  |  |  | 27.763 |  |  |  |  |  |  |
|  | 33.022 |  |  |  |  |  |  |  |  |  |  |  |  |  |  |
| 2C | 29.038 | 28.6665 | 22.868 |  | 5.7985 | -6.4735 | 88.862326 | 2L | 25.69 | 25.6853333 |  | 19.9 | 5.78533333 |  | 0.73052219 |
|  | 28.723 |  |  |  |  |  |  |  | 25.707 |  |  |  |  |  |  |
|  | 28.61 |  |  |  |  |  |  |  | 25.659 |  |  |  |  |  |  |
| 3C | 31.875 | 32.2075 | 21.261 |  | 10.9465 | -1.3255 | 2.50619731 | 3L | 29.496 | 29.2016667 |  | 21.438 | 7.76366667 | 2.43116667 | 0.18543687 |
|  | 32.091 |  |  |  |  |  |  |  | 29.138 |  |  |  |  |  |  |
|  | 32.324 |  |  |  |  |  |  |  | 28.971 |  |  |  |  |  |  |
| 4C | 23.69 | 23.6405 | 19.596 |  | 4.0445 | -8.2275 | 299.725911 | 4L | 23.633 | 23.919 |  | 18.861 | 5.058 | -0.7273333 | 1.65519363 |
|  | 23.591 |  |  |  |  |  |  |  | 24.164 |  |  |  |  |  |  |
|  | 23.967 |  |  |  |  |  |  |  | 23.96 |  |  |  |  |  |  |
| 5C | 24.394 | 24.8735 | 18.843 |  | 6.0305 | -6.2415 | 75.662157 | 5L | 24.454 | 24.454 |  | 17.891 | 6.563 | 1.2305 | 0.4248425 |
|  | 24.971 |  |  |  |  |  |  |  | 24.456 |  |  |  |  |  |  |
|  | 24.776 |  |  |  |  |  |  |  | 24.581 |  |  |  |  |  |  |
| 6C | 23.545 | 23.5433333 | 19.511 |  | 4.03233333 | -8.2396667 | 302.124632 | 6L | 23.87 | 23.827 |  | 17.714 | 6.113 | 0.7805 | 0.582165 |
|  | 23.583 |  |  |  |  |  |  |  | 23.837 |  |  |  |  |  |  |
|  | 23.502 |  |  |  |  |  |  |  | 23.774 |  |  |  |  |  |  |
| 7C | 26.598 | 26.6263333 | 20.16 |  | 6.46633333 | -5.8056667 | 55.7152361 | 7L | 24.761 | 24.6863333 |  | 18.736 | 5.95033333 | 0.61783333 | 0.65164885 |
|  | 26.683 |  |  |  |  |  |  |  | 24.645 |  |  |  |  |  |  |
|  | 26.598 |  |  |  |  |  |  |  | 24.653 |  |  |  |  |  |  |
| 8C | 23.891 | 23.7006667 | 15.494 |  | 8.20666667 | -4.0653333 | 16.7489645 | 8L | 24.936 | 24.8903333 |  | 17.898 | 6.99233333 | 1.65983333 | 0.31647571 |
|  | 23.537 |  |  |  |  |  |  |  | 24.817 |  |  |  |  |  |  |
|  | 23.674 |  |  |  |  |  |  |  | 24.918 |  |  |  |  |  |  |
| 9C | 26.231 | 26.2466667 | 19.902 |  | 6.34466667 | -5.9273333 | 62.682899 | 9L | 26.697 | 26.568 |  | 18.646 | 7.922 | 2.5895 | 0.1661433 |
|  | 26.203 |  |  |  |  |  |  |  | 26.583 |  |  |  |  |  |  |
|  | 26.306 |  |  |  |  |  |  |  | 26.424 |  |  |  |  |  |  |
| 10C | 24.638 | 24.86 | 16.403 |  | 8.457 | -3.815 | 14.0743852 | 10L | 27.273 | 27.3066667 |  | 18.491 | 8.81566667 | 3.48316667 | 0.0894257 |
|  | 24.869 |  |  |  |  |  |  |  | 27.528 |  |  |  |  |  |  |
|  | 25.073 |  |  |  |  |  |  |  | 27.119 |  |  |  |  |  |  |
| 11c | 26.755 | 26.71 | 20.352 |  | 6.358 | -5.914 | 60.2964015 | 11L | 29.373 | 29.412 |  | 19.539 | 9.873 | 4.5405 | 0.04297079 |
|  | 26.665 |  |  |  |  |  |  |  | 29.486 |  |  |  |  |  |  |
|  |  |  |  |  |  |  |  |  | 29.377 |  |  |  |  |  |  |
| 12C | 25.976 | 25.812 | 18.566 |  | 7.246 | -5.026 | 32.5819264 | 12L | 26.452 | 26.5985 |  | 18.271 | 8.3275 | 2.995 | 0.12543397 |
|  | 25.699 |  |  |  |  |  |  |  | 26.745 |  |  |  |  |  |  |
|  | 25.761 |  |  |  |  |  |  |  |  |  |  |  |  |  |  |
| 13C | 23.618 | 23.5866667 | 19.707 |  | 3.87966667 | -8.3923333 | 302.124632 | 13L | 26.463 | 26.787 |  | 19.169 | 7.618 | 2.2855 | 0.2051143 |
|  | 23.486 |  |  |  |  |  |  |  | 26.961 |  |  |  |  |  |  |
|  | 23.656 |  |  |  |  |  |  |  | 26.937 |  |  |  |  |  |  |
| 14C | 26.987 | 26.9453333 | 19.104 |  | 7.84133333 | -4.4306667 | 21.5557372 | 14L | 28.392 | 28.5476667 |  | 17.647 | 10.9006667 | 5.56816667 | 0.02107729 |
|  | 26.899 |  |  |  |  |  |  |  | 28.617 |  |  |  |  |  |  |
|  | 26.95 |  |  |  |  |  |  |  | 28.634 |  |  |  |  |  |  |
| 15C | 26.189 | 26.0723333 | 19.144 |  | 6.92833333 | -5.3436667 | 40.504211 | 15L | 31.914 | 31.939 |  | 17.877 | 14.062 | 8.7295 | 0.00235591 |
|  | 25.954 |  |  |  |  |  |  |  | 31.964 |  |  |  |  |  |  |
|  | 26.074 |  |  |  |  |  |  |  |  |  |  |  |  |  |  |
| 16C | 25.611 | 25.36 | 18.293 |  | 7.067 | -5.205 | 38.0546277 | 16L | 32.864 | 32.6553333 |  | 19.382 | 13.2733333 | 7.94083333 | 0.00406978 |
|  | 25.157 |  |  |  |  |  |  |  | 32.002 |  |  |  |  |  |  |
|  | 25.312 |  |  |  |  |  |  |  | 33.1 |  |  |  |  |  |  |
| 17C | 24.061 | 24.0826667 | 17.392 |  | 6.69066667 | -5.5813333 | 47.835176 | 17L | 26.509 | 26.1363333 |  | 19.253 | 6.88333333 | 1.55083333 | 0.34131286 |
|  | 24.231 |  |  |  |  |  |  |  | 25.898 |  |  |  |  |  |  |
|  | 23.956 |  |  |  |  |  |  |  | 26.002 |  |  |  |  |  |  |
| 18C | 25.077 | 24.9706667 | 18.856 |  | 6.11466667 | -6.1573333 | 71.357838 | 18L | 26.13 | 26.122 |  | 18.831 | 7.291 | 1.9585 | 0.25729583 |
|  | 24.817 |  |  |  |  |  |  |  | 26.114 |  |  |  |  |  |  |
|  | 25.018 |  |  |  |  |  |  |  | 25.634 |  |  |  |  |  |  |
| 19C | 26.715 | 26.5353333 | 19.902 |  | 6.63333333 | -5.6386667 | 49.7974513 | 19L | 29.899 | 29.686 |  | 20.65 | 9.036 | 3.7035 | 0.07676008 |
|  | 26.231 |  |  |  |  |  |  |  | 29.782 |  |  |  |  |  |  |
|  | 26.66 |  |  |  |  |  |  |  | 29.377 |  |  |  |  |  |  |
| 20C | 25.912 | 25.7936667 | 18.745 |  | 7.04866667 | -5.2233333 | 37.3568266 | 20L | 30.961 | 30.725 |  | 21.361 | 9.364 | 4.0315 | 0.06115016 |
|  | 25.516 |  |  |  |  |  |  |  | 30.56 |  |  |  |  |  |  |
|  | 25.953 |  |  |  |  |  |  |  | 30.654 |  |  |  |  |  |  |
| 21C | 25.364 | 25.25 | 22.388 |  | 2.862 | -9.41 | 680.287137 | 21L | 25.345 | 25.294 |  | 18.946 | 6.348 | 1.0155 | 0.49465686 |
|  | 25.039 |  |  |  |  |  |  |  | 25.294 |  |  |  |  |  |  |
|  | 25.347 |  |  |  |  |  |  |  | 25.243 |  |  |  |  |  |  |
| 22C | 25.23 | 25.4116667 | 17.875 |  | 7.53666667 | -4.7353333 | 26.6303595 | 22L | 25.389 | 25.3423333 |  | 18.142 | 7.20033333 | 1.86783333 | 0.27398459 |
|  | 25.241 |  |  |  |  |  |  |  | 25.365 |  |  |  |  |  |  |
|  | 25.764 |  |  |  |  |  |  |  | 25.273 |  |  |  |  |  |  |
| 23C | 30.629 | 30.3606667 | 27.616 |  | 2.74466667 | -9.5273333 | 737.756031 | 23L | 26.229 | 26.25 |  | 19.962 | 6.288 | 0.9555 | 0.51566284 |
|  | 29.994 |  |  |  |  |  |  |  | 26.178 |  |  |  |  |  |  |
|  | 30.459 |  |  |  |  |  |  |  | 26.343 |  |  |  |  |  |  |
| 24C | 24.169 | 24.1623333 | 18.817 |  | 5.34533333 | -6.9266667 | 121.600049 | 24L | 28.679 | 28.457 |  | 21.613 | 6.844 | 1.5115 | 0.35074635 |
|  | 24.24 |  |  |  |  |  |  |  | 28.408 |  |  |  |  |  |  |
|  | 24.078 |  |  |  |  |  |  |  | 28.284 |  |  |  |  |  |  |
| 25C | 25.659 | 25.4733333 | 17.724 |  | 7.74933333 | -4.5226667 | 22.9751122 | 25L | 27.553 | 27.1586667 |  | 19.311 | 7.84766667 | 2.51516667 | 0.17492802 |
|  | 25.516 |  |  |  |  |  |  |  | 27.121 |  |  |  |  |  |  |
|  | 25.245 |  |  |  |  |  |  |  | 26.802 |  |  |  |  |  |  |
| 26C | 25.164 | 25.115 | 19.117 |  | 5.998 | -6.274 | 77.3859633 | 26L | 26.227 | 26.246 |  | 19.129 | 7.117 | 1.7845 | 0.29027656 |
|  | 25.066 |  |  |  |  |  |  |  | 26.116 |  |  |  |  |  |  |
|  |  |  |  |  |  |  |  |  | 26.395 |  |  |  |  |  |  |
| 27C | 24.297 | 24.3886667 | 17.921 |  | 6.46766667 | -5.8043333 | 55.7152361 | 27L | 26.419 | 26.3253333 |  | 18.862 | 7.46333333 | 2.13083333 | 0.22832594 |
|  | 24.611 |  |  |  |  |  |  |  | 26.328 |  |  |  |  |  |  |
|  | 24.258 |  |  |  |  |  |  |  | 26.229 |  |  |  |  |  |  |
| 28C | 26.801 | 26.7403333 | 14.98 |  | 11.7603333 | -0.5116667 | 1.41421356 | 28L | 27.559 | 27.6123333 |  | 19.038 | 8.57433333 | 3.24183333 | 0.10570875 |
|  | 26.735 |  |  |  |  |  |  |  | 27.912 |  |  |  |  |  |  |
|  | 26.685 |  |  |  |  |  |  |  | 27.366 |  |  |  |  |  |  |
| 29C | 24.942 | 24.9486667 | 17.439 |  | 7.50966667 | -4.7623333 | 27.1397087 | 29L | 25.833 | 25.7963333 |  | 18.181 | 7.61533333 | 2.28283333 | 0.20549379 |
|  | 24.87 |  |  |  |  |  |  |  | 25.742 |  |  |  |  |  |  |
|  | 25.034 |  |  |  |  |  |  |  | 25.814 |  |  |  |  |  |  |
